# Psychometric assessment of scales used to evaluate sexual assault prevention programming in the United States Air Force

**DOI:** 10.1371/journal.pone.0317557

**Published:** 2025-01-16

**Authors:** Kathryn E. L. Grimes, Nichole M. Scaglione, Marni L. Kan, Leah Frerichs, Christopher M. Shea, Angela M. Stover

**Affiliations:** 1 RTI International, Health Practice Area, Research Triangle Park, North Carolina, United States of America; 2 Department of Health Policy and Management, Gillings School of Global Public Health, University of North Carolina at Chapel Hill, Chapel Hill, North Carolina, United States of America; 3 Department of Health Education and Behavior, College of Health and Human Performance, University of Florida, Gainesville, Florida, United States of America; 4 Lineberger Comprehensive Cancer Center, Chapel Hill, North Carolina, United States of America; Tribhuvan University Institute of Medicine, NEPAL

## Abstract

**Background:**

Preventing sexual assault in the United States (U.S.) military is essential to safeguard the overall well-being of military personnel and support the military to function in alignment with its intended mission and objectives. Valid instruments are needed to accurately and reliably evaluate programming effectiveness. The goal of this research was to psychometrically assess measures used to evaluate the Sexual Communication and Consent (SCC) program within the Air Force Basic Military Training (BMT) context.

**Methods:**

We evaluated four measures used to assess the SCC program implemented at Air Force BMT in 2019–2020: Date Rape Attitudes, Self-Efficacy to Resist Unwanted Advances, Risky and Protective Dating Behaviors, and Bystander Intentions. The analytic sample included 7,126 BMT trainees (74% male). We assessed structural validity with exploratory factor analysis (EFA) and confirmatory factor analysis (CFA). We used full information maximum likelihood estimation with robust standard errors in Mplus. We refined each scale based on factor analysis results and assessed internal consistency reliability by computing Cronbach’s coefficient alpha for each scale in the overall sample, by sex, and by tailored SCC intervention group.

**Results:**

We identified a two-factor structure for the Date Rape Attitudes scale; subscale reliability was moderate within the overall sample and among males, though low among females. We found single-factor structures and excellent reliability for both the Self-Efficacy to Resist Unwanted Advances scale and the Bystander Intentions scale. The Dating Behaviors scale CFA did not confirm the two-factor solution suggested by the EFA, but subscale reliability was acceptable.

**Conclusion:**

This research fills a critical gap in the psychometric literature in military settings. Based on our findings, we recommend approaches for using the finalized scales in future evaluations of sexual assault prevention programming in the U.S. Air Force BMT setting.

## Introduction

The Department of Defense (DoD) estimated 29,061 active duty service members in the United States (U.S.) military experienced unwanted sexual contact in 2023 [1]. Prevalence varied across gender and military service branch, with figures ranging from 4.6% to 10.8% among women and 0.8% to 1.8% among men [1]. The rates of sexual victimization are higher over the course of one’s military career: a meta-analysis estimated 15.7% (3.9% percent of men and 38.4% of women) experienced sexual harassment or assault during military service [2]. Addressing sexual assault is essential given its detrimental impact on both the individual and organizational levels. The harmful physical and psychological outcomes related to sexual assault have been extensively documented and include injury, sexually transmitted infection, pregnancy, depression, anxiety, self-harm, and post-traumatic stress disorder [3,4]. On an organizational level, sexual assault affects personnel performance and retention, unit cohesion, and readiness [5–8]. Preventing sexual assault is essential to safeguard the overall well-being of military personnel and support the U.S. military to function in alignment with its intended mission and objectives.

The DoD aims to foster a military culture devoid of sexual assault and harassment, as these behaviors contradict military core values and undermine the required trust to build and sustain a fighting force [1,9]. To accomplish this goal, the military must invest in effective prevention programming to address and mitigate these harmful behaviors. However, research on the effectiveness of prevention programming in the military is scarce. A recent systematic review revealed that just five sexual assault prevention programs have been evaluated in the military [10]. The study populations in this review only included members of either the Army or the Navy and the programs evaluated did not represent the breadth of prevention programming that was being implemented across diverse military settings [10]. In addition, while a range of outcome scales were leveraged to evaluate program effectiveness, none of the studies explored scale validity within military populations. To the authors’ knowledge, no measures assessing the effectiveness of sexual assault prevention programs in the U.S. military have been validated specifically for use in military populations.

Those responsible for implementing sexual assault prevention programming must have valid instruments to measure intervention effectiveness. Higher-level sexual assault prevalence data is helpful for indicating broader trends; however, it cannot identify the aspects of the intervention that contribute to meaningful change. The Sexual Assault Prevention and Response (SAPR) FY21-FY25 Research Agenda includes a research priority to “identify and validate indicators of sexual assault prevention and response outcomes for use in prevention and response planning, program, and policy evaluation.” [11]. Accurately reporting evidence-based insights is essential to build trust and credibility in programming among interest holders, including military personnel and policymakers. Measure validation will contribute to accuracy and reliability in future evaluations, suggest ways to streamline scales to promote parsimony and alleviate survey fatigue, and facilitate the ability for the military to compare findings more confidently across different military contexts. Ultimately, robust evaluation methods are fundamental to advancing the overall goal of reducing sexual assault incidence within the military to foster a safer and more supportive environment for all service members.

The goal of this research was to psychometrically assess measures used to evaluate sexual assault prevention programming within the Air Force Basic Military Training (BMT) context. The specific objectives of this research were to examine structural validity and internal consistency reliability to finalize measures for future evaluation of sexual assault prevention programs implemented in the BMT setting.

## Methods

### Setting & participants

The Sexual Communication and Consent (SCC) program was implemented at Lackland Air Force Base in San Antonio, Texas between September 2019 and March 2020 among BMT trainees. SCC was adapted from four evidence-based programs that have demonstrated effectiveness in school settings [12–15]. Trainees received one of five tailored, electronically-delivered SCC interventions depending on how they responded to a screening instrument assessing individual sexual assault risk [16]: Female Revictimization Prevention, Female Primary Victimization Prevention, Male Revictimization Prevention, Male Primary Victimization Prevention, or Healthy Relationships/Bystander Intervention.

BMT is an 8.5-week long bootcamp to prepare individuals to become enlisted members of the U.S. Air Force. Approximately 20,000–30,000 individuals complete BMT in a typical year [17]. During the implementation period, the SCC program replaced standard sexual assault prevention instruction at BMT for a total of 6 hours of training content across two days: Day 1 SCC occurred on week 2 of BMT and Day 2 of SCC occurred in week 4 of BMT. Due to substantial Day 1 missingness on measures of interest, the current study used the sample of 7,126 BMT trainees who received Day 2 SCC training. This research was approved by the research ethics committees of the researchers’ institutions and the DoD Office of Human Research Oversight. The study was given a non-human subjects research designation because sexual assault prevention training is a BMT requirement, therefore, trainees did not undergo informed consent. However, before the training began, we informed trainees of the steps taken to protect their privacy and emphasized that responding to survey questions was completely voluntary.

### Data sources & measures

SCC outcomes were measured in the pre- and post-training surveys that trainees completed electronically on tablets before and after participating in the SCC program (S1 Appendix). Measures were primarily sourced from the four evidence based programs adapted to develop SCC training content [12–15]. Source measures reflected commonly targeted constructs in sexual assault prevention. Original program developers shared the measures they used to evaluate their programs, along with the source scales from which those measures were derived, granting permission to adapt and use them to evaluate the SCC program. In adapting measures for SCC, we reduced scale length to mitigate participant burden and adapted measures to be gender neutral and specific to an Air Force context. Further refinements to each scale were necessary to ensure the pre- and post-training surveys could effectively evaluate all five tailored SCC interventions while keeping the survey length brief and balanced for all trainees. We prioritized including the scale items that were used to evaluate the original programs used to develop SCC and those most relevant to SCC intervention content, as including items unrelated to training content could mask program effects. We cognitively tested the adapted scales in June 2017 among 6 male and 3 female Airmen who had recently completed BMT. The objectives of the cognitive testing were to: (1) test the wording changes and determine if the content and language used was relevant to an Air Force population, (2) ensure items were written at the appropriate reading level and that Airmen interpreted them in the way they were intended to be interpreted, and (3) ensure the survey was an appropriate length and level of burden. Results led to further refining and clarifying language in items across scales before SCC implementation.

Trainees received different measures in the pre- and post-training surveys based on the tailored SCC program to which they were assigned. All trainees answered questions on date rape attitudes (Table 1). Trainees completing the Healthy Relationships/Bystander Intervention answered questions on bystander intentions to intervene. Trainees completing the Victimization Prevention and Revictimization Prevention programs answered questions related to self-efficacy to resist unwanted advances related to sexual assault victimization and risky and protective dating behaviors.

**Table 1:** Outcome measures collected from trainees via tablet in pre- and post-training surveys.

SCC Program	Measure	# Items	Instructions	Sample Item	Response Options	Source Scales Informing Measures
All trainees	Date Rape Attitudes	10	The next questions are about your opinions. There are no right or wrong answers. Please indicate the degree to which you disagree or agree with the following statements.	*If someone is unsure about whether they want sex*, *it is okay for their partner to persist until they flatly say no*.	1 – Strongly disagree 2 – Disagree 3 – Neither agree or disagree 4 – Agree 5 – Strongly agree	Date Rape Attitudes Scale [13] Rape Attitudes and Beliefs Scale (RABS)[18]
Primary Victimization or Revictimization Prevention	Self-Efficacy to Resist Unwanted Advances	6	For these items, please indicate your level of confidence in how you would respond to the following situations.	*How confident are you that you could…successfully recognize the signs that you might be in danger of being sexually assaulted*?	1 – Not at all confident 2 3 4 – Moderately confident 5 6 7 – Extremely confident	Self-Efficacy Ratings Scale [12,19]
	Risky and Protective Dating Behaviors	15 (female); 14 (male)	Please indicate the choice which best describes your typical behavior on the first few dates.	*On the first few dates…I let a friend or family member know where I am and whom I am with*	1 – Never 2 – Rarely 3 – Some of the time 4 – About half of the time 5 – Most of the time 6 – Always	Dating Behavior Survey [14] Dating Self-Protection Against Rape Scale (DSPARS) [20] Original items created for this study based on intervention content
Healthy Relationships / Bystander Intervention	Bystander Intentions	11	Based on the scale provided, indicate how likely you are to engage in the following intervening behaviors	*How likely is it that you would…intervene if you saw a man hitting on a woman and she appeared not to want it*?	1 – Not at all likely 2 3 4 5 – Extremely likely	Intentions to Intervene [13] Reactions to Offensive Language and Behavior (ROLB) [21] Bystander Attitude Scale [22] Bystander Efficacy Scale [22]

#### Sociodemographic data

Sociodemographic information collected from trainees with the screening instrument included self-identified sex (male; female), sexual orientation (heterosexual or straight; gay or lesbian; bisexual; another identity [e.g., questioning, asexual, undecided, self-identified]), and relationship status (exclusive romantic relationship; non-exclusive romantic relationship; not in a romantic relationship). Per U.S. Air Force policy, we did not collect additional sociodemographic information such as age, race, or ethnicity to protect trainee confidentiality.

#### Date rape attitudes

We used 10 items to measure date rape attitudes among all SCC participants (S1 Appendix). We adapted our measure from the 17-item date rape attitudes measure Salazar et al (2014) used to evaluate a web-based bystander intervention among college undergraduate men [13]. Items were sourced from the 50-item Rape Attitudes and Beliefs Scale (RABS)[18], which was originally developed for use among college men. Burgess (2007) reported the original RABS had a five-factor structure with Cronbach’s alphas across subscales ranging from 0.73 to 0.85. Nine of our 10 Date Rape Attitudes items came from the RABS and one item was developed for this study based on intervention content. We modified wording to be gender neutral and Air Force-specific, to define terms, and to simplify language. Items represented stereotyped or prejudicial beliefs about sexual assault, such as “*When it comes to sex*, *women say no when they mean yes to avoid seeming ‘too easy*.*’*” Respondents indicated their level of agreement with each item, and higher scores represented greater acceptance of date rape attitudes. As an attention check, two of 10 items did not reflect date rape attitudes (e.g., “*Rape can occur between two Airmen–even if they seem to be a normal couple who are often seen together at parties*”) and therefore were reverse scored.

#### Self-efficacy to resist unwanted advances

We used six items to measure self-efficacy to resist unwanted advances related to sexual assault victimization among participants assigned the Revictimization Prevention and Primary Victimization Prevention programs (S1 Appendix). We adapted our measure from the seven-item Self-Efficacy Ratings scale used to evaluate a program to reduce women’s risk for sexual victimization [12,19]. Marx et al. (2001) did not report the Self-Efficacy Ratings scale’s psychometric properties, however, a recent application of the Self Efficacy Ratings scale among college women reported Cronbach’s alpha of 0.85 [23]. We rephrased all items from Marx et al. (2001) to use the same question stem and ensure all items were gender neutral. Items included scenarios that required resisting unwanted advances, such as confidence in avoiding situations in which one could be sexually assaulted or resisting someone’s pressures to drink alcohol. Participants indicated their level of confidence with each item, and higher scores represented greater confidence to resist unwanted advances related to sexual assault victimization.

#### Risky and protective dating behaviors

We used 15 items to measure risky and protective dating behaviors among participants in the Revictimization Prevention and Primary Victimization Prevention programs who reported they had ever dated someone (S1 Appendix). We adapted our measure from the 15-item Dating Behavior Survey [14] and the 15-item Dating Self-Protection Against Rape Scale (DSPARS) [20]. The Dating Behavior Survey had a one-week test-retest reliability of 0.77 and a Cronbach’s alpha of 0.63 among a sample of 350 female college students [14]. The DSPARS had a Cronbach’s alpha of 0.86 and a Spearman-Brown reliability coefficient of 0.81 among a sample of 120 male and female college students [20]. We used 11 items from the Dating Behavior Survey and five items from the DSPARS, selected for relevance to BMT and SCC training content. We did not include items that used outdated language or were not clearly a risk or protective behavior. We made minor grammatical changes to facilitate comprehension, terminology changes for consistency, and used gender neutral pronouns to be more widely applicable to both male and female respondents. Items included behaviors such as consuming alcohol or other drugs, meeting in public or private settings, and providing one’s own transportation. Respondents indicated the frequency with which they engaged in these behaviors when with a new dating partner. Higher scores represented higher frequency engaged in that risk behavior when with a new dating partner. Nine of 15 items reflected protective strategies (e.g., “*I let a friend or family member know where I am and whom I am with*”) and therefore were reverse scored.

#### Bystander intentions

We used 11 items to measure bystander intentions to intervene among participants assigned the Healthy Relationships/Bystander Intervention program (S1 Appendix). We adapted our measure from the 15-item scale Salazar et al. (2014) used to measure Intentions to Intervene, which they adapted from the Reactions to Offensive Language and Behavior (ROLB) index, the Bystander Efficacy scale, and the Bystander Attitude scale [13,21,22]. Salazar et al. (2014) reported a Cronbach’s alpha of 0.94 in their sample of 743 male undergraduate students [13]. We did not include items that were not applicable to SCC intervention content (e.g., *“Express my discomfort if a professor makes an offending remark”*) and made minor changes to items from Salazar et al. to improve readability and remain grammatically consistent with updated question stems. Items included various scenarios warranting bystander intervention, such as “*How likely is it that you would…Intervene if you saw a man hitting on a woman and she appeared to not want it*.” Higher scores represented greater likelihood to intervene as a bystander. Three items reflected bystander inaction (e.g., *“Say nothing if you heard your friends tell sexist jokes*.”) and were therefore reverse scored.

### Data analysis strategy

All analyses were performed using Stata version 17.0 [24] and Mplus version 8.10 [25]. To finalize the sample, we dropped observations that had duplicate identification codes recorded on the same day (n = 94), as this prevented us from accurately distinguishing individual participants. We also dropped those who took less than two minutes to complete the pre- or post-training survey (n = 667); as trainees were asked to respond to between 62–71 survey items depending on the SCC program to which they were assigned, we used a conservative estimate of two seconds per question to identify potentially carelessly invalid responses that could be producing nonrandom error [26,27]. After finalizing the sample, we conducted descriptive analyses to understand sociodemographic characteristics of the population as well as item- and scale-level missingness in the pre-training and post-training data. This preliminary missingness assessment revealed substantial scale missingness in the pre-training data; therefore, we used post-training data to conduct psychometric assessments on outcome measures.

We performed factor analyses to explore and then confirm structural validity. Post-training data (N = 7,126) were randomly split into two samples: one sample for exploratory factor analysis (EFA) (N = 3,564) and one sample for confirmatory factor analysis (CFA) (N = 3,562). Chi-square comparisons confirmed the split samples did not significantly differ from each other by self-identified sex or SCC intervention assignment. In both the EFA and CFA models, we used full information maximum likelihood estimation (FIML) with robust standard errors (MLR estimation) to adjust for missing values and nonnormal distributions. Treating ordinal data with at least five response categories as continuous does not significantly impact the accuracy of factor analysis results, and using MLR allows for better handling of missing data and for the generation of reliable fit indices [28,29].

We first conducted an EFA for each scale to identify a parsimonious solution that best reproduced observed correlations in the data. We used oblique rotation (geomin) to allow the underlying factors to correlate. We followed Kaiser’s rule and retained factors if eigenvalues were greater than 1 and examined scree plots to verify that we were not over factoring [30]. We sought a simple structure for each factor, whereby each item loaded meaningfully onto only one factor and the factor loadings on other factors were trivial or close to zero [31,32]. We considered factor loadings ≥0.30 as meaningful and ≥0.40 as strong, and required each factor have at least three non-cross loading items to be retained [31,33,34]. Model finalization was an iterative process that included examining how eigenvalues and factor loadings changed when non-vocal items or poorly defined factors were excluded. The research team assessed factor interpretability to ensure factors had distinct conceptual meaning.

For CFA models, the a priori guidelines to assess model fit followed recommendations from Hu and Bentler (1999), which included the Comparative Fit Index (CFI), Root Mean Square Error of Approximation (RMSEA), and Standardized Root Mean Residual (SRMR) [35]. We considered CFI values ≥0.90 and ≥0.95 as suggesting acceptable and excellent fit, respectively. For RMSEA and SRMR, we considered values ≤0.08 and ≤0.06 to indicate acceptable and excellent fit, respectively. If the model reached the cutoffs identified in two out of the three descriptive fit indices (RMSEA, CFI, SRMR) we determined the model had adequate fit. As the likelihood ratio chi-square is highly influenced by sample size we did not consider this statistic for model fit [36]. For completeness we reported the Satorra-Bentler Scaled chi-square (S-B *χ*^2^) in lieu of the non-scaled chi-square test statistic as the data demonstrated statistically significant (p<0.01) multivariate skewness [37,38].

After refining scales based on factor analysis results, we assessed internal consistency reliability by computing Cronbach’s coefficient alpha (α) for each scale in the overall post-training sample (N = 7,126), by self-identified sex, and by SCC intervention assignment.

## Results

### Descriptive analyses

Sample statistics are shown in Table 2. Data were available for 7,126 trainees in the post-training period after dropping observations that had same-day unique identification code duplicates and those who took less than two minutes to complete the survey. The majority of the sample was male (74.4%), identified as heterosexual or straight (91.6%), and not in a romantic relationship (51.1%). Two-thirds of trainees (65.8%) were assigned to the Male Healthy Relationships / Bystander Intervention SCC program, 16.7% of trainees were assigned the Female Primary Victimization prevention program, and 8.9% of trainees were assigned the Female Revictimization Prevention intervention. The lowest proportion of trainees were assigned Male Revictimization Prevention and Male Primary Victimization Prevention programs, representing 5.5% and 3.0% of the overall sample, respectively.

**Table 2:** Sociodemographic characteristics and intervention assignment, post-training sample.

Characteristic	Post-Training	
	(N = 7,126)	
	n	%
Sex		
Male	5,301	74.4
Female	1,825	25.6
Sexual Orientation†		
Heterosexual or straight	5,204	91.6
Gay or lesbian	120	2.1
Bisexual	287	5.1
Questioning/asexual/undecided	71	1.3
Relationship Status†		
Exclusive romantic relationship	2,590	45.4
Non-exclusive romantic relationship	200	3.5
Not in a romantic relationship	2,913	51.1
Intervention assignment		
Female Revictimization Prevention	637	8.9
Female Primary Victimization Prevention	1,188	16.7
Male Revictimization Prevention	394	5.5
Male Primary Victimization Prevention	216	3.0
Male Healthy Relationships / Bystander Intervention	4,691	65.8

^†^Missing: Sexual orientation (n = 1,444), relationship status (n = 1,423)

### Date rape attitudes

#### EFA

The EFA ran with 2,871 observations, which represented 80.6% (2,871/3,564) of the sample eligible to respond to Date Rape Attitudes scale in the EFA dataset. Factor loadings in the one-factor model were meaningful for all items (≥0.30) except for Item 8 (“*It is rare for women to say they have been raped simply because they feel guilty about having sex*”), which had a factor loading of -0.13. This item was also non-vocal in the two-, three-, and four-factor models. In addition, the three- and four-factor models did not meet the criteria of having at least three items load strongly onto each factor; therefore, we did not consider the three- or four-factor models as appropriate for this dataset. We removed Item 8 and reran the EFA.

In the EFA that excluded Item 8, a two-factor solution best fit the data and explained 59.7% of the total variance (S2 Appendix). We named the first factor Consent-Related Assumptions and the second factor Rape Myths and Misconceptions. Geomin rotated factor loadings onto Consent Related Assumptions ranged from 0.46 to 0.78 and factor loadings onto Rape Myths and Misconceptions ranged from 0.39 to 0.89 (Table 3).

**Table 3:** EFA and CFA results for the final 8-item date rape attitudes scale.

Item	EFA Geomin rotated factor loadings		CFA Standardized Factor Loadings	
	Consent-Related Assumptions	Rape Myths and Misconceptions	Consent-Related Assumptions	Rape Myths and Misconceptions
1. If someone is unsure about whether they want sex, it is okay for their partner to persist until they flatly say no.	**0.46** *	0.18*	0.64**	-
2. It is okay to have sex with someone who is drunk.	**0.57** *	0.16*	0.72**	-
3. If two people willingly go to some private or secluded place (such as one of their rooms), they intend to have sex.	**0.78** *	0.00	0.71**	-
4. In many cases, if someone is raped by an acquaintance (someone they know), the person who was raped has to take some responsibility for what happened.	0.28*	**0.40** *	-	0.67**
5. Rape can occur between two Airmen–even if they seem to be a normal couple who are often seen together at parties.†	-0.18*	**0.39** *	-	[Dropped]
6. Both men and women alike see activities like kissing, touching, and fondling as a sign sexual intercourse is going to happen.	**0.47** *	-0.08	0.39**	-
7. If a person wants to increase their chances of having sex, they should get the other person drunk.	0.11*	**0.74** *	-	0.86**
9. When it comes to sex, women say no when they mean yes to avoid seeming "too easy."	0.02	**0.78** *	-	0.81**
10. It is okay to have sex with someone who doesn’t clearly communicate they want to have sex, as long as they aren’t visibly resisting. (Note: communication can be verbal or non-verbal)	-0.03	**0.89** *	-	0.85**

^†^Denotes reverse-scored item.

*Indicates factor loading is significant at p<0.05;

**indicates factor loading is significant at p<0.01.

*Note*. The final Date Rape Attitudes scale excludes Items 8 and 5: EFA results exclude Item 8, and CFA results exclude both Item 8 and Item 5.

#### CFA

The CFA ran with 2,883 observations, which represented 80.9% (2,883/3,562) of the sample eligible to respond to Date Rape Attitudes scale in the CFA dataset. While results demonstrated that a two-factor model fit well for the nine-item Date Rape Attitudes scale (S-B *χ*^2^ [1] = 137.09, p<0.01; RMSEA = 0.05; CFI = 0.96; SRMR = 0.03), the standardized factor loading for Item 5 (“*Rape can occur between two Airmen–even if they seem to be a normal couple who are often seen together at parties*”) was only 0.27 and therefore did not meet the a priori threshold for meaningful factor loading. This low factor loading suggested removing this item would improve the scale. Due to the item’s complexity and its unique use of the Air Force-specific term "Airman" we ultimately excluded this item from our proposed final Date Rape Attitudes scale.

The final 8-item Date Rape Attitudes scale ran with 2,882 observations and supported a two-factor structure (S-B *χ*^2^ [1] = 129.73, p<0.01; RMSEA = 0.05; CFI = 0.97; SRMR = 0.03). Standardized factor loadings for Consent-Related Assumptions subscale ranged from 0.39 to 0.72 and standardized factor loadings for Rape Myths and Misconceptions subscale ranged from 0.67 to 0.85 (Table 3). All standardized factor loadings were statistically significant (p<0.01). The two factors were highly correlated at 0.83 (standard error [SE]: 0.017) and statistically significant (p<0.01), suggesting a strong relationship between the Consent-Related Assumptions and Rape Myths and Misconceptions subscales.

#### Internal consistency reliability

The Consent-Related Assumptions 4-item subscale had moderate reliability for the overall sample (α = 0.71) (Cronbach’s alphas and item-rest correlations for final scales and subscales can be found in S3 Appendix). When exploring reliability by self-identified sex, reliability was higher for males (α = 0.72) compared to females (α = 0.60). Internal consistency was lowest among those assigned the Female Revictimization Prevention SCC program (α = 0.57) and highest among those assigned the Healthy Relationships/Bystander Intervention program (α = 0.74).

Reliability for the Rape Myths and Misconceptions 4-item subscale was strong for the overall sample (α = 0.86). Reliability was higher for males (α = 0.88) compared to females (α = 0.75). Alpha was lowest among those assigned the Female Revictimization Prevention program (α = 0.74) and highest within the Healthy Relationships/Bystander Intervention program (α = 0.89).

### Self-efficacy to resist unwanted advances

#### EFA

The EFA model ran with 990 observations, which represented 81.3% (990/1,218) of the sample eligible to respond to the Self Efficacy to Resist Unwanted Advances scale in the EFA dataset. The EFA suggested a one-factor solution best explained the data. The first factor had an eigenvalue of 4.29 and explained 71.5% of the total variance (S2 Appendix). No other eigenvalues exceeded 1.0. Geomin rotated factor loadings ranged from 0.65–0.93 (Table 4).

**Table 4:** EFA and CFA results for the final 6-item self-efficacy to resist unwanted advances scale.

Item	EFA Geomin rotated factor loadings	CFA Standardized Factor Loadings
1. Successfully resist someone’s advances if they were attempting to get you to have sex and you were not interested?	0.79*	0.78**
2. Tell someone that you would pay for your own way if they were attempting to pay for your meal when you did not want them to?	0.65*	0.62**
3. Successfully resist someone’s pressuring if they were attempting to get you to consume alcohol, despite your wishes not to do so?	0.67*	0.67**
4. Successfully avoid a situation in which you could be sexually assaulted?	0.88*	0.90**
5. Successfully think up ways to get out of a situation and execute your plan, if a situation develops in which you feel you could be in danger of sexual assault?	0.93*	0.89**
6. Successfully recognize the signs that you might be in danger of being sexually assaulted?	0.90*	0.83**

*Indicates factor loading is significant at p<0.05;

**indicates factor loading is significant at p<0.01

*Note*. This table presents the full 6-item Self-Efficacy Ratings scale.

#### CFA

The CFA ran with 954 observations, which represented 78.4% (954 /1,217) of the sample eligible to respond to Self-Efficacy Ratings scale in the CFA dataset. Results demonstrated that a one-factor model fit well for the six-item Self Efficacy to Resist Unwanted Advances scale (S-B *χ*^2^ [1] = 37.41, p<0.01; RMSEA = 0.11; CFI = 0.90; SRMR = 0.06). All standardized factor loadings were large (0.62 to 0.90) and statistically significant (p<0.01), supporting the one-factor structure (Table 4).

#### Internal consistency reliability

Internal consistency reliability for the final six-item scale was excellent for the overall sample (α = 0.91) (S3 Appendix). When exploring reliability by self-identified sex, reliability was consistent for both males (α = 0.91) and females (α = 0.91). Cronbach’s alpha remained consistent across SCC intervention subgroups, ranging from α = 0.91 to α = 0.92.

### Risky and protective dating behaviors

#### EFA

One of the 15 items in the Risky and Protective Dating Behaviors scale was only asked of female participants (Item 11) and was therefore not included in the factor analysis. The EFA ran with 830 observations, which represented 92.0% (830/902) of the sample eligible to respond to the Risky and Protective Dating Behaviors scale in the EFA dataset. Factor loadings on the one-factor model were only meaningful for five of 14 items and therefore did not fit the data well. In the two-factor model, Item 2 (“*My date and I do things that allow us to spend time alone together*”) and Item 5 (“*I pay for my own expenses*”) were non-vocal. In addition, the three- and four-factor models had issues with factor loadings >1.0 suggesting overspecification and did not meet the requirement of having at least 3 items meaningfully load onto a factor to retain; therefore, we did not consider the three- or four-factor models as appropriate for this dataset. We removed Items 2 and 5 and reran the EFA.

In the EFA that excluded Items 2, 5, and 11, a two-factor solution best fit the data and explained 57.9% of the total variance (S2 Appendix). We named the first factor Risky Behaviors and the second factor Protective Behaviors. Geomin rotated factor loadings onto Risky Behaviors ranged from 0.59 to 0.89 and geomin rotated factor loadings onto Protective Behaviors ranged from 0.43–0.78 (Table 5).

**Table 5:** EFA and CFA results for the final 12-item risky and protective dating behaviors scale.

	EFA Geomin rotated factor loadings		CFA Standardized Factor Loadings	
	Risky Behaviors	Protective Behaviors	Risky Behaviors	Protective Behaviors
1. I consume alcohol or other drugs	**0.83** *	-0.11*	0.91**	-
3. My date consumes alcohol or other drugs	**0.86** *	-0.09*	0.92**	-
4. I consume enough alcohol or other drugs to become drunk or high	**0.89** *	0.03	0.85**	-
6. My date consumes enough alcohol or other drugs to become drunk or high	**0.87** *	0.007	0.84**	-
7. I provide my own transportation or carry enough money in case I need to get myself home later (e.g., for a bus, taxi, etc.)†	-0.14*	**0.43** *	-	0.37**
8. My date and I choose group activities (e.g., double date or spend time with friends) †	-0.03	**0.39** *	-	0.36**
9. I have "blacked out" from alcohol or other drugs (lose consciousness, can’t remember what happened)	**0.59** *	0.06	0.55**	-
10. Before I go out with someone for the first time, I try to find out about them†	0.03	**0.67** *	-	0.52**
12. I pay attention to my date’s alcohol or other drug intake†	-0.007	**0.62** *	-	0.57**
13. My date and I meet in public places (e.g., a restaurant) †	-0.03	**0.66** *	-	0.66**
14. I let a friend or family member know where I am and whom I am with†	0.002	**0.78** *	-	0.71**
15. In general, I plan for what self-protective measures I would take if I were alone with my date and they became sexually aggressive†	0.04	**0.76** *	-	0.73**

^†^Denotes reverse-scored item.

*Indicates factor loading is significant at p<0.05;

**indicates factor loading is significant at p<0.01.

*Note*. While the original Dating Behaviors scale had 15 items, male respondents skipped one item (Item 11) so we consider the full original scale for this research to be 14 items. This table presents the Dating Behaviors scale excluding Items 2, 5, and 11.

#### CFA

The CFA ran with 818 observations, which represented 91.4% (818/895) of the sample eligible to respond to the Risky and Protective Dating Behaviors scale in the CFA dataset. Results did not confirm a two-factor structure for the final 12-item Risky and Protective Dating Behaviors scale (S-B *χ*^2^ [2] = 412.94, p<0.01; RMSEA = 0.09; CFI = 0.85; SRMR = 0.05). Standardized factor loadings were all statistically significant (p<0.01) and ranged from 0.55 to 0.92 for Risky Behaviors and 0.36 to 0.73 for Protective Behaviors (Table 5). The two factors had a weak but significant correlation at 0.13 (SE: 0.05, p<0.01).

To determine what subscale may have contributed to less than acceptable fit in the two-factor CFA, we ran a one-factor CFA on the Risky Behaviors subscale and a one-factor CFA on the Protective Behaviors subscale. The five-item Risky Behaviors CFA ran with 818 observations (91.4% of sample eligible to respond) and did not confirm a one-factor structure (S-B *χ*^2^ [1] = 33.11, p<0.01; RMSEA = 0.15; CFI = 0.87; SRMR = 0.05), though standardized factor loadings were high (0.54 to 0.92) and statistically significant (p<0.01). The seven-item Protective Behaviors CFA ran with 774 observations (86.5% of sample eligible to respond) and confirmed a one-factor structure (S-B *χ*^2^ [1] = 0.25, p = 0.62; RMSEA = 0.07; CFI = 0.95; SRMR = 0.04). Standardized factor loadings were all statistically significant (p<0.01) and ranged from 0.36 to 0.75.

#### Internal consistency reliability

The Risky Dating Behaviors 5-item subscale had excellent reliability (α = 0.90) for the overall sample of participants with dating history assigned Primary Victimization or Revictimization Prevention programs (S3 Appendix). Reliability was slightly higher for males with dating history (α = 0.92) compared to females with dating history (α = 0.89). Cronbach’s alpha ranged from 0.89 (Female Primary Victimization Prevention) to 0.93 (Healthy Relationships/ Bystander Intervention).

The Protective Dating Behaviors 7-item subscale had acceptable reliability (α = 0.78) for the overall sample of participants with dating history assigned Primary Victimization or Revictimization Prevention program (S3 Appendix). Reliability was higher for females with dating history (α = 0.80) compared to males with dating history (α = 0.75). Cronbach’s alpha ranged from 0.75 (Male Revictimization and Male Primary Victimization) to 0.81 (Female Revictimization Prevention) across SCC intervention groups.

### Bystander intentions

#### EFA

The EFA model ran with 1,995 observations, which represented 85.0% (1,995/2,346) of the sample eligible to respond to the Bystander Intentions scale in the EFA dataset. The one-factor model had strong geomin rotated factor loadings (ranging from 0.70 to 0.88) for all items except the three reverse-scored items, which had factor loadings ranging from -0.008 to 0.09. In the two-factor model, these three reverse-scored items all meaningfully loaded onto a second factor, however, upon reviewing each item’s wording we concluded this was a product of how the items were phrased and not reflective of a distinct construct. The three- and four-factor models had issues with items being multivocal and factors lacked at least three non-cross-loading items; the three- and four-factor models were therefore disregarded.

After dropping the three reverse-scored items, a one-factor model best fit the data. The first factor had an eigenvalue of 5.40 and explained 67.5% of total variance (S2 Appendix). No other eigenvalues exceeded 1.0. Geomin rotated factor loadings in the one-factor model were strong and ranged from 0.67 to 0.88 (Table 6).

**Table 6:** EFA and CFA results for the final 8-item bystander intentions scale.

Item	EFA Geomin rotated factor loadings	CFA Standardized Factor Loadings
1. Intervene if you saw a man hitting on a woman and she appeared not to want it	0.80*	0.80**
2. Intervene if you witnessed a situation in which it looked like a woman might end up being taken advantage of	0.88*	0.88**
3. Express discomfort if you heard a man/group of men using bad language or offensive names when talking about women	0.67*	0.67**
4. Intervene if you saw a man hitting on a woman who appeared to be extremely intoxicated	0.87*	0.86**
6. Express your discomfort if someone said that rape victims are to blame for being raped	0.77*	0.71**
7. Ask a friend if they needed to be walked or driven home from a party	0.70*	0.71**
8. Do something to help a drunk person who was being taken upstairs to a bedroom at a party	0.84*	0.86**
10. Intervene if you saw a friend taking a very intoxicated person up the stairs to his/her room	0.82*	0.83**

*Indicates factor loading is significant at p<0.05;

**indicates factor loading is significant at p<0.01

*Note*. This table presents the Bystander Intentions scale excluding Items 5, 9, and 11.

#### CFA

The CFA ran with 2,013 observations, which represented 85.8% (2,013/2,345) of the sample eligible to respond to the Bystander Intentions scale in the CFA dataset. Results demonstrated that a one-factor model fit well for the eight-item Bystander Intentions scale (S-B *χ*^2^ [1] = 3.09, p = 0.08; RMSEA = 0.10; CFI = 0.91; SRMR = 0.05). All standardized factor loadings were large, ranging from 0.67 to 0.88 and statistically significant (p<0.01), supporting the one-factor structure (Table 6).

#### Internal consistency reliability

Internal consistency was excellent (α = 0.93) for trainees assigned the Male Healthy Relationships / Bystander Intervention SCC program (S3 Appendix).

## Discussion

### Summary of findings

The goal of this study was to psychometrically evaluate scales assessing sexual assault prevention programming for a military population. Specifically, we calculated structural validity and internal consistency reliability to refine four measures to evaluate the Sexual Communication and Consent (SCC) program implemented at Air Force BMT in 2019–2020: Date Rape Attitudes, Self-Efficacy to Resist Unwanted Advances, Risky and Protective Dating Behaviors, and Bystander Intentions. We identified a two-factor structure for the Date Rape Attitudes scale, a one-factor structure for the Self-Efficacy to Resist Unwanted Advances and Bystander Intentions scales, and mixed findings for the Risky and Protective Dating Behaviors scale (see S4 Appendix for a complete summary of results). Below we compare our findings of structural validity and internal consistency reliability to the literature, recommend how to use the finalized scales and subscales, and discuss implications for future research.

#### Date rape attitudes

Based on factor analysis results, we removed two items to reduce the original 10-item Date Rape Attitudes scale to eight items. The CFA confirmed a two-factor structure for the final eight-item Date Rape Attitudes scale (four items in each subscale). We named the two subscales Consent-Related Assumptions and Rape Myths and Misconceptions. These subscales were highly correlated (0.83), indicating that while they represent distinct conceptual domains, they both closely reflect underlying attitudes toward date rape. This high correlation suggests that an individual who scores highly on one subscale is likely to score highly on the other.

Psychometric research on the Rape Attitudes and Beliefs Scale (RABS) is in its nascency compared to other, more widely used scales such as the Illinois Rape Myth Acceptance Scale (IRMAS) [39] and the Rape Myth Acceptance Scale (RMAS) [40]. While the RABS has been less widely adopted, Burgess (2007) cited the outdated or colloquial language and notable lack of context in previously developed scales as justification for developing the RABS [18]. Burgess identified five subscales–Status, Tactics, Gender, Justifications, and Blame–in their EFA among 368 male college students. However, the eight Date Rape Attitudes items we retained did not load onto the same subscales as in the original study. The four items in our Consent-Related Assumptions subscale loaded onto the Tactics, Status, and Justifications subscale in Burgess’s study. Similarly, the four items in our Rape Myths and Misconceptions subscale loaded onto the Tactics, Status, and Justifications subscales.

The structural validity Burgess reported for the original 50-item RABS has yet to be replicated in other studies. In a replication study among 225 college students, Briones (2009) retained only 36 of the original 50 items and found that several items loaded onto different subscales than those reported by Burgess; they renamed the subscales Not Rape, Coercion, Gender Role, Misinterpretation, and Sexual Power [41]. Similarly, Hays et al. (2016) used 39 of the original 50 items and ultimately treated subscales independently due to low explained variance and lack of unidimensionality when they conducted an EFA with the full 50-item RABS among a sample of male and female college students [42]. Burgess retained multivocal items that had reasonable factor loadings onto more than one factor, which may have contributed to difficulties in replicating findings. For instance, the item *“In many cases*, *if a woman is raped by an acquaintance*, *she has to take some responsibility for what happened to her”* had factor loadings of 0.48 and 0.41 onto the Justifications and Blame subscales, respectively [18]. This item loaded onto Burgess’s subscale they titled Not Rape, and in our study this item loaded onto the Rape Myths and Misperceptions subscale. Inconsistent findings across studies suggests that date rape attitudes are difficult to define, complex, and can vary substantially across different contexts. More psychometric research is needed to better understand the validity of the RABS in various populations.

Internal consistency reliability was lower for females than males in this sample. This finding is not surprising, as the RABS was originally developed for and tested among men [18]. Cronbach’s alpha for the two subscales in this research among males in this sample (α = 0.72 and α = 0.88) was comparable to the alphas reported for each of the five RABS subscales, which ranged from 0.73 to 0.85 [18]. Reliability for the Rape Myths and Misconceptions subscale within our sample of male BMT trainees was also consistent with the measure Salazar et al (2014) used in their evaluation of the RealConsent web-based bystander intervention among college undergraduate men, which used 17 items from the RABS and reported a Cronbach’s alpha of 0.86 [13]. This is the first study to our knowledge to report internal consistency reliability among a sample of female participants (α = 0.60 and α = 0.75).

*Recommendations*. Based on the low internal consistency reliability among females in this sample, we recommend using the Consent-Related Assumptions and Rape Myths and Misconceptions subscales only among males in the Air Force BMT context. This recommendation is in line with previous research, which has concluded attitudinal measures may not be appropriate tools to measure sexual assault prevention program effectiveness among women because attitudes have not been shown to predict sexual victimization among women [43]. Additionally, the high correlation between these subscales suggests future analyses could create a single composite Date Rape Attitudes score for simplicity or take a multivariate approach to assess subscale changes while accounting for high correlation.

#### Self-efficacy to resist unwanted advances

The final Self-Efficacy to Resist Unwanted Advances scale remained unchanged from the original six-item measure. This analysis confirmed a one-factor structure. To our knowledge, this study is the first to measure the validity of the Self-Efficacy Ratings scale. We adapted our measure from the Self-Efficacy Ratings Scale used by Marx et al (2001), who adapted their measure from Ozer and Bandura’s (1990) Self-Defense Self-Efficacy scale [12,19]. In a principal components analysis, Ozer and Bandura identified a one-factor structure for 12 items and reported factor loadings from 0.55 to 0.95 [19,44,45]. However, Ozer and Bandura’s psychometric analysis of the Self Defense Self-Efficacy scale is not directly comparable to this research based on the stark differences in study populations: their study was conducted among 43 women in the San Francisco Bay area who enrolled in a self-defense program and captured responses on a 10-point scale (0 = complete uncertainty; 10 = complete certitude) [19]. Literature on structural validity of self-efficacy scales to evaluate sexual assault prevention programming is scarce and we were unable to identify any psychometric studies exploring the structural validity of Self-Efficacy measures used to evaluate sexual assault prevention programming.

Internal consistency reliability was high among the overall sample (α = 0.91) and remained consistent for males (α = 0.91) and females (α = 0.91). Although this is lower than the reliability coefficient of 0.97 reported by Ozer & Bandura (1990), internal consistency in this Air Force trainee sample is higher than recent studies reporting internal consistency reliability of the Self-Efficacy Ratings scale among college students, where alphas ranged from 0.80 to 0.85 [23,46,47].

*Recommendations*. The one-factor structure and high internal consistency reliability among both males and females in our sample suggests the final six-item Self Efficacy to Resist Unwanted Advances scale is appropriate to use in future evaluations of sexual assault prevention programing in the Air Force BMT context.

#### Risky and protective dating behaviors

The original Risky and Protective Dating Behaviors scale included 15 items. We removed three items throughout factor analyses and proposed a final 12-item Risky and Protective Dating Behaviors scale that had two subscales. We named the first factor Risky Behaviors and the second factor Protective Behaviors. While the EFA suggested a two-factor structure, the CFA did not confirm this finding because it did not meet the cutoff criteria for at least two out of the three descriptive fit indices (RMSEA, CFI, SRMR) established a priori. However, descriptive fit thresholds were almost reached (e.g., CFI = 0.85 and we designated values of ≥0.90 as acceptable fit), suggesting that our findings may not hold within a different sample. Independent one-factor CFAs revealed the Risky Behaviors subscale did not have a one-factor structure; however, the CFA supported a one-factor structure within the seven-item Protective Behaviors subscale.

It is unclear why the CFA did not confirm a two-factor structure. While not presented in this paper due to page constraints, the item-level analysis for the final Risky Behavior items demonstrated substantial skewness. This skewness ranged from 1.26 to 3.71, with response options 5 and 6 representing “Most of the time” and “Always” (Table 1) were rarely selected, potentially resulting from social desirability bias. For instance, across Risky Behavior items, the proportion of respondents selecting “Always” ranged from 0.06% to 0.34% of the sample. This lack of variability across response categories in the Risky Behaviors subscale may have contributed to poor performance from the maximum likelihood estimator. In a study examining the relative performance of robust categorical least squares estimation (cat-LS) compared to robust continuous maximum likelihood estimation (ML), researchers found that cat-LS outperformed ML in circumstances with less than five response categories [28]. In addition, ML estimators had difficulty when observed variables had “extreme category asymmetry” [28]. Considering the distributions in our data, six response options may be too many categories; future studies may want to consider taking an Item Response Theory (IRT) approach to determine whether collapsing categories into fewer response options is necessary.

Items in the Risky Behaviors scale were sourced from the Dating Behaviors Survey and items from the Protective Behaviors subscale were predominantly sourced from the Dating Self-Protection Against Rape scale (DSPARS) [14,20]. We found a weak yet significant correlation between the Risky Behaviors and Protective Behaviors subscales in our analysis. This finding was unexpected, as previous investigations into the convergent validities of the source scales identified items in the DSPARS were negatively correlated with risk-related dating behaviors [43]. In exploring the nonparametric Spearman correlations within the Dating Behaviors scale, only one item in the Protective Behaviors subscale was negatively correlated with any items in the Risk Behaviors subscale: *“I provide my own transportation or carry enough money in case I need to get myself home later*.*”* It is possible that individuals in our sample who engaged in more dating behaviors overall might report both risk and protective behaviors due to more frequent and varied interactions, which could be driving this positive correlation.

Studies reporting the structural validity of the Dating Behaviors Survey and the DSPARS are limited. We were able to identify just one study that reported the structural validity of the Dating Behavior survey using principal components analysis (PCA), which did not reveal a strong factor structure [48]. However, this analysis conducted by Davis et al (2006) had limited generalizability, in that only six of the 14 items were from the original Dating Behaviors Survey and two-thirds of their study sample was excluded from the PCA [48]. While the DSPARS developers reported initial construct validity, we were unable to find studies reporting the structural validity of this measure.

Internal consistency reliability was high in the overall eligible sample for the Risky Behaviors subscale (α = 0.90) and was consistent for males (α = 0.92) and females (α = 0.89). These findings are higher than the reliability estimates of the Dating Behaviors Survey in the literature, which ranged from 0.63 to 0.71 among college women [49,50].

Internal consistency reliability was respectable in the overall eligible sample for the Protective Behaviors subscale (α = 0.78). Alpha was slightly higher among females (α = 0.80) compared to males (α = 0.75). These findings are consistent with reliability estimates of the DSPARS in the literature, which ranged from 0.79 to 0.86 among college students [47,50,51].

*Recommendations*. CFA findings did not confirm the two-factor structure identified in the EFA and the independent CFAs on each subscale revealed issues stemmed from the Risk Behaviors subscale. For this reason–and to reduce survey length and participant burden–we recommend only using the seven-item Protective Behaviors subscale in future evaluations of sexual assault prevention programming in the Air Force BMT context. In addition, due to lack of variability and skewness in participant responses, future Dating Behaviors scale development should consider taking an IRT approach to determine if fewer response options are needed.

#### Bystander intentions

Based on factor analysis results, we removed three reverse scored items from the original 11-item Bystander Intentions scale. The final eight-item measure had a one factor structure and excellent internal consistency reliability (α = 0.93).

Our Bystander Intentions scale sourced items from Salazar et al (2014), who used the Reactions to Offensive Language and Behavior (ROLB) index, the Bystander Efficacy Scale, and the Bystander Attitude scale [21,22]. The 26-item ROLB index was conceptualized–not confirmed with factor analyses–to have four subscales [21]. To our knowledge, the ROLB has not yet been validated via factor analysis in any population. Banyard et al. (2005) indicated that all measures developed for their study were “pilot tested for reliability and validity prior to their use” [22], however, to our knowledge, the only psychometric data reported in the literature for the Bystander Efficacy and Bystander Attitudes scale include internal consistency reliability and pre- to post-test correlation [22,52,53]. In a recent systematic review to identify validated measures related to bystander intervention, Mennicke et al. (2023) identified 16 scales related to interpersonal violence, of which nine pertained specifically to bystander attitudes or intentions [54]. Scale validation methods across these nine studies varied and all were conducted among samples of either college students (77.8%) or high school students (22.2%) [54], highlighting the urgent need for psychometric assessments of bystander measures in more diverse populations.

Internal consistency reliability was high in the sample of males assigned the Healthy Relationships/ Bystander Intervention SCC program (α = 0.93). This is consistent with reliability estimates previously reported for the ROLB, Bystander Efficacy Scale, and Bystander Attitudes scales (α = 0.79–0.99) [13,21,22].

*Recommendations*. Our findings of a one-factor structure and high internal consistency reliability within our male sample suggest the final eight-item Bystander Intentions scale is appropriate to use in future evaluations of sexual assault prevention programming among males in the Air Force BMT context.

### Future research implications

More research is needed to evaluate the psychometric properties of measures used to assess sexual assault prevention programming effectiveness in diverse populations, particularly within the military. There is a notable gap in the literature regarding validity of these measures, especially within military populations. While researchers commonly report reliability using Cronbach’s alpha, this metric has several limitations, including privileging scales with more items, the assumption of unidimensionality, and potential bias with ordinal data [55]. Conducting a CFA is an alternative means to estimate scale reliability that addresses these limitations and also assesses scale dimensionality [31]. Although multiple group factor analysis was beyond the scope of this research, further testing structural validity of these measures across various identities (e.g., gender, sexual orientation, race, ethnicity) within a military sample is warranted. Multiple group CFA will ensure measures are being interpreted similarly across groups and increase generalizability of findings within a context [31]. This research took a Classical Test Theory approach [56]; however, future research could benefit from the strengths of Item Response Theory (IRT). IRT offers a more nuanced understanding of item performance, helps identify overlapping response categories, and tests item bias through differential item functioning across subpopulations [57]. Finally, qualitative research could enhance our understanding of how military populations interpret items on these finalized scales. Future studies should use cognitive interviewing techniques to understand how this population comprehends questions, standardize response options, and verify items are not offensive to survivors.

### Limitations

Data missingness was the primary limitation of this study, the cause of which was partially known. Through observations during SCC implementation, we discovered several instructors cut the amount of time allocated for the survey on Day 1 short; we emphasized the importance of providing enough time for trainees to complete the survey to instructors, which may have explained the reduced missingness on Day 2. Additionally, many trainees likely felt sleep-deprived and overwhelmed from demanding BMT duties during SCC implementation, which may have exacerbated survey fatigue. Missingness increased for each scale as the scale progressed and across the entire instrument, suggesting survey length contributed to nonresponse. Given the data did not appear to be missing at random, we were unable to perform multiple imputation to address data missingness. Further, a full information maximum likelihood (FIML) approach treating data as continuous was preferred to using weighted least squares mean and variance adjusted (WLSMV) estimator with categorical data, as the latter approach uses listwise deletion, which can bias estimates [28].

Conducting psychometric analyses on survey measures post hoc may affect results interpretation. We were unable to adjust the survey measures based on psychometric findings, which may have impacted the quality and accuracy of findings. As there is such a substantial gap in the literature on the validity and reliability of survey measures for evaluating sexual assault prevention interventions in the U.S. military this research still makes a substantial contribution to the literature. However, future studies should plan to optimize measures during the initial design phase prior to implementation to reduce potential measurement error.

Our findings are generalizable to trainees in the Air Force BMT context. The large sample size increases our confidence that results are applicable to the overall trainee population. Further research is needed to support scale validity and reliability in different military branches and populations (e.g., cadets, active-duty service members) before leveraging these scales in various military contexts.

## Conclusion

This research assessed the psychometric properties of four measures to evaluate sexual assault prevention programs in the Air Force BMT context. Not only does this research fill a critical gap in the psychometric literature in military settings, but it is also in line with the FY21-FY25 SAPR research agenda to validate indicators measuring sexual assault prevention programming. Conducting high quality research on sexual assault prevention programming effectiveness requires psychometrically assessed measurement tools. With valid and reliable scales, we can better evaluate interventions aimed at reducing sexual assault and fostering a safe environment within the military.

## Supporting information

S1 Appendix Original pre- and post-training survey used in the sexual communication and consent (SCC) program implementation at U.S. Air Force Basic Military Training (BMT) 2019–2020. (DOCX)

S2 Appendix Exploratory factor analysis eigenvalues and scree plots. (DOCX)

S3 Appendix Internal consistency reliability overall, by self-identified sex, and by SCC program assignment for final scales. (DOCX)

S4 Appendix Summary of EFA, CFA, and internal consistency reliability results. (DOCX)
