# Supplementary material for: Psychometric assessment of scales used to evaluate sexual assault prevention programming in the United States Air Force
Source: PLoS One. 2025 Jan 16;20(1):e0317557. doi: 10.1371/journal.pone.0317557 (PMC11737684; doi:10.1371/journal.pone.0317557)
Supplement: S1 Appendix — (DOCX) [file pone.0317557.s001.docx]

# S1 Appendix – Original Pre- and Post-Training Survey Used in Sexual Communication and Consent (SCC) Program Implementation at U.S. Air Force Basic Military Training (BMT) 2019-2020

Red text denotes skip patterns

*Indicates reverse coded items

## **Date Rape Attitudes**

***Date Rape Attitudes (all trainees)***

SOURCE: Salazar et al. (2014) adapted from the Rape Attitudes and Beliefs Scale (RABS)(Burgess, 2007)

The next questions are about **your opinions**. There are no right or wrong answers. Please indicate the degree to which you disagree or agree with each of the following statements.

|  | **Strongly disagree** | **Disagree** | **Neither disagree nor agree** | **Agree** | **Strongly agree** |
| --- | --- | --- | --- | --- | --- |
| 1. If someone is unsure about whether they want sex, it is okay for their partner to persist until they flatly say *no.* | 1 | 2 | 3 | 4 | 5 |
| 1. It is okay to have sex with someone who is drunk. | 1 | 2 | 3 | 4 | 5 |
| 1. It is an unspoken rule that if two people willingly go to some private or secluded place (such as one of their rooms), they intend to have sex. | 1 | 2 | 3 | 4 | 5 |
| 1. In many cases, if someone is raped by an acquaintance (someone they know), the person who was raped has to take some responsibility for what happened. | 1 | 2 | 3 | 4 | 5 |
| 1. Rape can occur between two Airmen—even if they seem to be a normal couple who are often seen together at parties. * | 1 | 2 | 3 | 4 | 5 |
| 1. Both men and women alike see activities like kissing, touching, and fondling as a sign sex is going to happen. |  |  |  |  |  |
| 1. If a person wants to increase their chances of having sex, they should get the other person drunk. | 1 | 2 | 3 | 4 | 5 |
| 1. It is rare for women to say they have been raped simply because they feel guilty about having sex. * | 1 | 2 | 3 | 4 | 5 |
| 1. When it comes to sex, women say *no* when they mean *yes* to avoid seeming “too easy.” | 1 | 2 | 3 | 4 | 5 |
| 1. It is okay to have sex with someone who doesn’t clearly communicate they want to have sex, as long as they aren’t visibly resisting.   (Note: communication can be verbal or nonverbal) | 1 | 2 | 3 | 4 | 5 |

## **Self-Efficacy to Resist Unwanted Advances**

***Self-Efficacy to Resist Unwanted Advances (Revictimization and Primary Victimization trainees only)***

SOURCE: Marx et al. (2001), adapted from Self-Defense Self-Efficacy (Ozer & Bandura, 1990)

For these items, please indicate your level of confidence in how you would respond to the following situations.

| **How confident are you that you could…?** | **Not at all Confident** | | **Moderately Confident** | | | **Extremely Confident** | |
| --- | --- | --- | --- | --- | --- | --- | --- |
| 1. Successfully resist someone’s advances if they were attempting to get you to have sex and you were not interested? | 1 | 2 | 3 | 4 | 5 | 6 | 7 |
| 1. Tell someone that you would pay for your own way if they were attempting to pay for your meal when you did not want them to? | 1 | 2 | 3 | 4 | 5 | 6 | 7 |
| 1. Successfully resist someone’s pressuring if they were attempting to get you to consume alcohol, despite your wishes not to do so? | 1 | 2 | 3 | 4 | 5 | 6 | 7 |
| 1. Successfully avoid a situation in which you could be sexually assaulted? | 1 | 2 | 3 | 4 | 5 | 6 | 7 |
| 1. Successfully think up ways to get out of a situation and execute your plan, if a situation develops in which you feel you could be in danger of sexual assault? | 1 | 2 | 3 | 4 | 5 | 6 | 7 |
| 1. Successfully recognize the signs that you might be in danger of being sexually assaulted? | 1 | 2 | 3 | 4 | 5 | 6 | 7 |

## **Risky and Protective Dating Behaviors**

***Dating Behaviors – Protective Strategies & Risk Factors (Revictimization and Primary Victimization trainees only)***

SOURCE: Dating Behavior Survey (Hanson & Gidycz, 1993); Dating Self-Protection Against Rape Scale (DSPARS) (Moore & Waterman, 1999)

1. Have you **ever** dated someone?

(Note: Dating includes spending time with someone you’re interested in, alone or with a group of people. It can include formal “dates” or informal activities, like hanging out at someone’s house, going to a movie or out to dinner with a group of friends, etc.)

- 1. Yes
  2. No

1. [SHOW IF Date = Yes] The next few questions are focused on your dating behaviors. Please think of how you are when you are with a new dating partner.

Please indicate the choice which best describes your **typical behavior** **on the first few dates** with someone.

| **On the first few dates…** | **Never** | **Rarely** | **Some of the time** | **About half of the time** | **Most of the time** | **Always** |
| --- | --- | --- | --- | --- | --- | --- |
| 1. I consume alcohol or other drugs | 1 | 2 | 3 | 4 | 5 | 6 |
| 1. My date and I to do things that allow us to spend time alone together (e.g., in one of our homes or rooms) | 1 | 2 | 3 | 4 | 5 | 6 |
| 1. My date consumes alcohol or other drugs | 1 | 2 | 3 | 4 | 5 | 6 |
| 1. I consume enough alcohol or other drugs to become drunk or high | 1 | 2 | 3 | 4 | 5 | 6 |
| 1. I pay for my own expenses * | 1 | 2 | 3 | 4 | 5 | 6 |
| 1. My date consumes enough alcohol or other drugs to become drunk or high | 1 | 2 | 3 | 4 | 5 | 6 |
| 1. I provide my own transportation or carry enough money in case I need to get myself home later (e.g., for a bus, taxi, etc.) * | 1 | 2 | 3 | 4 | 5 | 6 |
| 1. My date and I choose group activities (e.g., double date or spend time with friends) * | 1 | 2 | 3 | 4 | 5 | 6 |
| 1. I have "blacked out" from alcohol or other drugs (lose consciousness, can't remember what happened) | 1 | 2 | 3 | 4 | 5 | 6 |
| 1. Before I go out with someone for the first time, I try to find out about them * | 1 | 2 | 3 | 4 | 5 | 6 |
| 1. **SKIP IF MALE:** I stop dating someone if I realize they have negative attitudes towards women or they make sexist remarks* | 1 | 2 | 3 | 4 | 5 | 6 |
| 1. I pay attention to my date’s alcohol or other drug intake * | 1 | 2 | 3 | 4 | 5 | 6 |
| 1. My date and I meet in public places (e.g., a restaurant) * | 1 | 2 | 3 | 4 | 5 | 6 |
| 1. I let a friend or family member know where I am and whom I am with * | 1 | 2 | 3 | 4 | 5 | 6 |
| 1. In general, I plan for what self-protective measures I would take if I were alone with my date and they became sexually aggressive. * | 1 | 2 | 3 | 4 | 5 | 6 |

## **Bystander Intentions**

***Intention to Intervene (Healthy Relationships / Bystander Intervention trainees only)***

SOURCE: Salazar et al. (2014), adapted from the Reactions to Offensive Language and Behavior (ROLB) index (Loh et al., 2005), the Bystander Attitude Scale, and the Bystander Efficacy Scale (Banyard et al., 2005).

Based on the scale provided, indicate **how likely you are to engage** in the following **intervening behaviors**.

To **intervene** means helping someone who is in danger by asking if everything is okay, creating a diversion, directly addressing the issue or calling 911; expressing disapproval for offending remarks or behavior; stopping a person from doing something harmful to themselves or to another person; etc.

| **How likely is it that you would…?** | **Not at all Likely** |  |  |  | **Extremely Likely** |
| --- | --- | --- | --- | --- | --- |
| 1. Intervene if you saw a man hitting on a woman and she appeared not to want it | 1 | 2 | 3 | 4 | 5 |
| 1. Intervene if you witnessed a situation in which it looked like a woman might end up being taken advantage of | 1 | 2 | 3 | 4 | 5 |
| 1. Express discomfort if you heard a man/group of men using bad language or offensive names when talking about women | 1 | 2 | 3 | 4 | 5 |
| 1. Intervene if you saw a man hitting on a woman who appeared to be extremely intoxicated | 1 | 2 | 3 | 4 | 5 |
| 1. Do nothing if you saw a man being verbally abusive toward a woman* | 1 | 2 | 3 | 4 | 5 |
| 1. Express your discomfort if someone said that rape victims are to blame for being raped |  |  |  |  |  |
| 1. Ask a friend if they needed to be walked or driven home from a party | 1 | 2 | 3 | 4 | 5 |
| 1. Do something to help a drunk person who is being taken upstairs to a bedroom at a party | 1 | 2 | 3 | 4 | 5 |
| 1. Do nothing if you saw a woman looking very uncomfortable while surrounded by a group of men at a party* | 1 | 2 | 3 | 4 | 5 |
| 1. Intervene if you saw a friend taking a very intoxicated person up the stairs to his/her room | 1 | 2 | 3 | 4 | 5 |
| 1. Say nothing if you heard your friends tell sexist jokes* | 1 | 2 | 3 | 4 | 5 |

## **References**

Banyard, V. L., Plante, E. G., & Moynihan, M. M. (2005). *Rape Prevention Through Bystander Education: Bringing a Broader Community Perspective to Sexual Violence Prevention. Final report to NIJ for grant 2002-WG-BX-0009.* https://doi.org/10.1037/e535792006-001

Burgess, G. H. (2007). Assessment of Rape-Supportive Attitudes and Beliefs in College Men: Development, Reliability, and Validity of the Rape Attitudes and Beliefs Scale. *Journal of Interpersonal Violence*, *22*(8), 973–993. https://doi.org/10.1177/0886260507302993

Hanson, K. A., & Gidycz, C. A. (1993). Evaluation of a Sexual Assault Prevention Program. *Journal of Consulting and Clinical Psychology*, *61*(6), 1046–1052. https://doi.org/10.1037//0022-006x.61.6.1046

Loh, C., Gidycz, C. A., Lobo, T. R., & Luthra, R. (2005). A Prospective Analysis of Sexual Assault Perpetration: Risk Factors Related to Perpetrator Characteristics. *Journal of Interpersonal Violence*, *20*(10), 1325–1348. https://doi.org/10.1177/0886260505278528

Moore, C. D., & Waterman, C. K. (1999). Predicting self-protection against sexual assault in dating relationships among heterosexual men and women, gay men, lesbians, and bisexuals. *Journal of College Student Development*, *40*(2), 132–140.

Ozer, E. M., & Bandura, A. (1990). Mechanisms Governing Empowerment Effects: A Self-Efficacy Analysis. *Journal of Personality and Social Psychology*, *58*(3), 472–486.
