# Supplementary material for: Psychometric assessment of scales used to evaluate sexual assault prevention programming in the United States Air Force
Source: PLoS One. 2025 Jan 16;20(1):e0317557. doi: 10.1371/journal.pone.0317557 (PMC11737684; doi:10.1371/journal.pone.0317557)

# S2 Appendix. Exploratory Factor Analysis Eigenvalues and Scree Plots

## Date Rape Attitudes

Table 1. Eigenvalues for the sample correlation matrix, Date Rape Attitudes EFA

|  | Full 10-item Scale | | | 9-item Scale  (After Removing Item 8) | | |
| --- | --- | --- | --- | --- | --- | --- |
| Factor | Eigenvalue | Variance explained by factor | Total variance explained by solution | Eigenvalue | Variance explained by factor | Total variance explained by solution |
| 1 | 4.28 | 42.77% | 42.77% | 4.26 | 47.32% | 47.32% |
| 2 | 1.18 | 11.83% | 54.60% | 1.12 | 12.40% | 59.72% |
| 3 | 0.93 | 9.34% | 63.94% | 0.76 | 8.44% | 68.17% |
| 4 | 0.76 | 7.59% | 71.53% | 0.67 | 7.46% | 75.62% |
| 5 | 0.66 | 6.59% | 78.12% | 0.60 | 6.63% | 82.26% |
| 6 | 0.59 | 5.93% | 84.05% | 0.56 | 6.22% | 88.48% |
| 7 | 0.56 | 5.59% | 89.64% | 0.44 | 4.91% | 93.39% |
| 8 | 0.44 | 4.42% | 94.06% | 0.33 | 3.69% | 97.08% |
| 9 | 0.33 | 3.31% | 97.37% | 0.26 | 2.92% | 100.00% |
| 10 | 0.26 | 2.63% | 100.00% |  |  |  |
| Sum | 10.00 |  |  | 9.00 |  |  |

Figure 1. Scree Plot, Date Rape Attitudes EFA, full 10-item scale


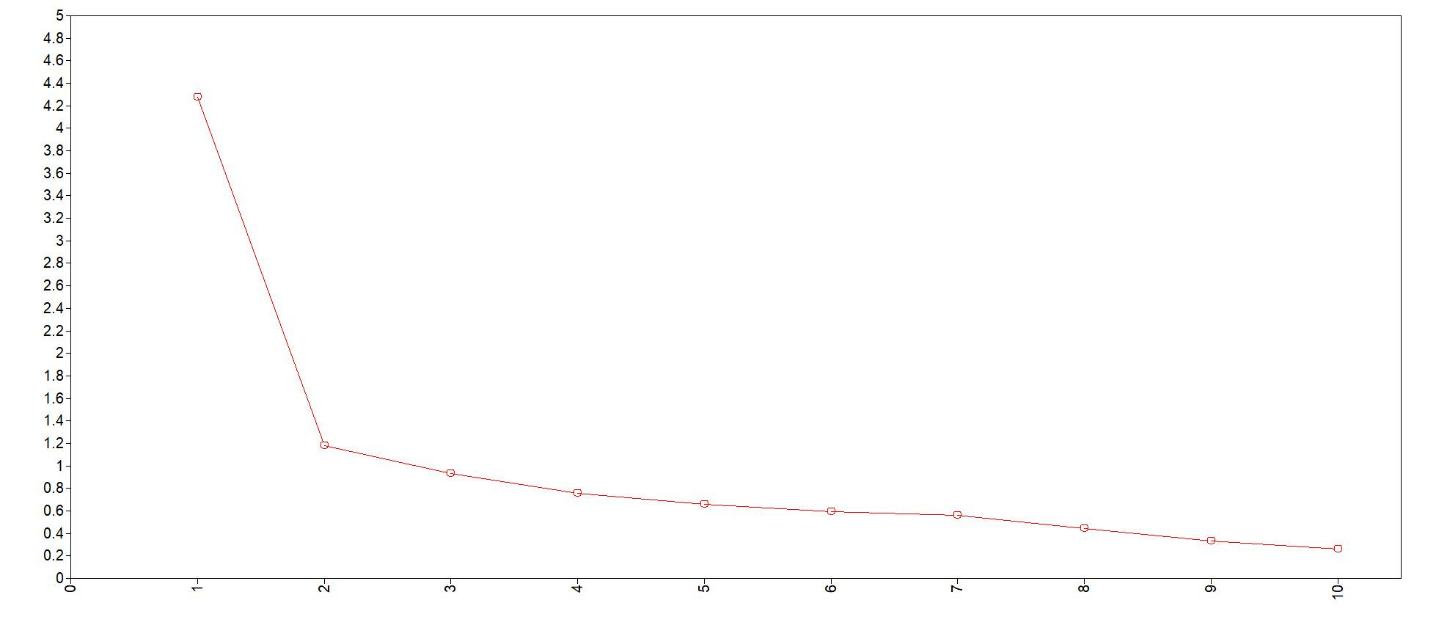


Figure 2. Scree Plot, Date Rape Attitudes EFA, 9-item scale after removing Item 8


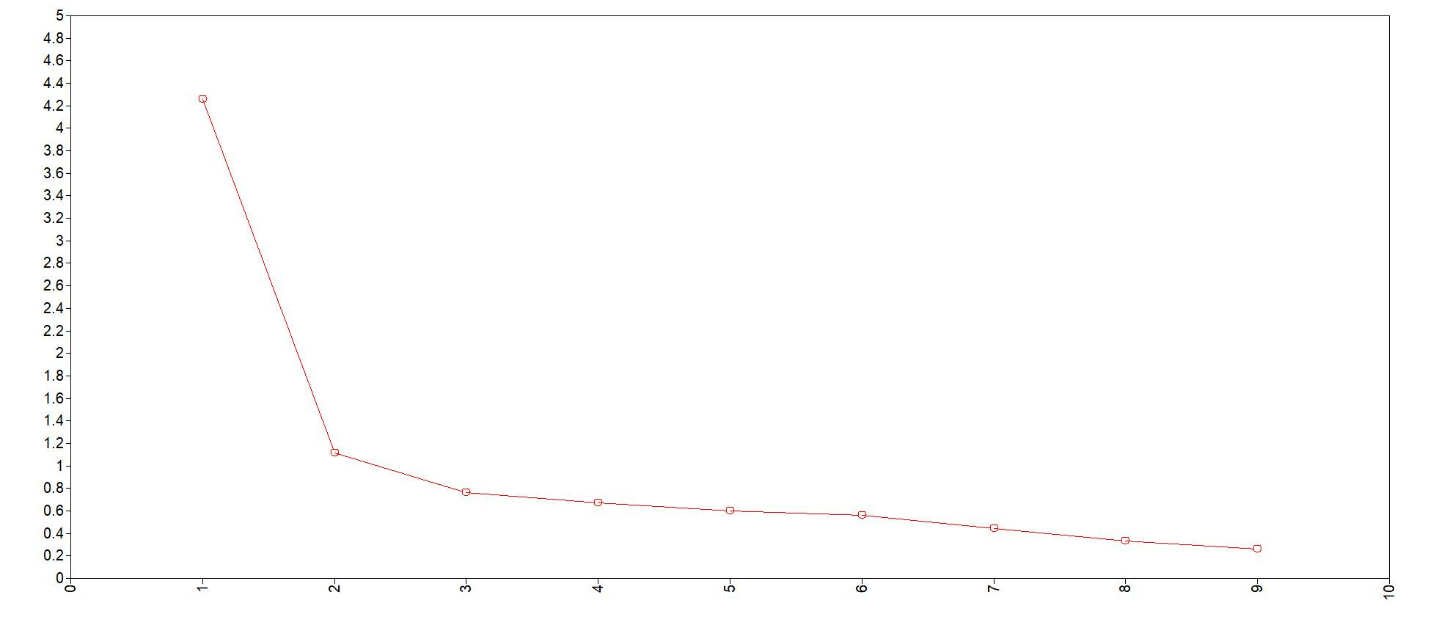


## Self-Efficacy to Resist Unwanted Advances

Table 2. Eigenvalues for sample correlation matrix, Self-Efficacy to Resist Unwanted Advances EFA, full 6-item scale

| Factor | Eigenvalue | Variance explained by factor | Total variance explained by solution |
| --- | --- | --- | --- |
| 1 | 4.29 | 71.53% | 71.53% |
| 2 | 0.68 | 11.32% | 82.85% |
| 3 | 0.36 | 6.05% | 88.90% |
| 4 | 0.35 | 5.78% | 94.68% |
| 5 | 0.19 | 3.08% | 97.77% |
| 6 | 0.13 | 2.23% | 100.00% |
| Sum | 6.00 |  |  |

Figure 3. Scree plot, Self-Efficacy to Resist Unwanted Advances EFA, full 6-item scale


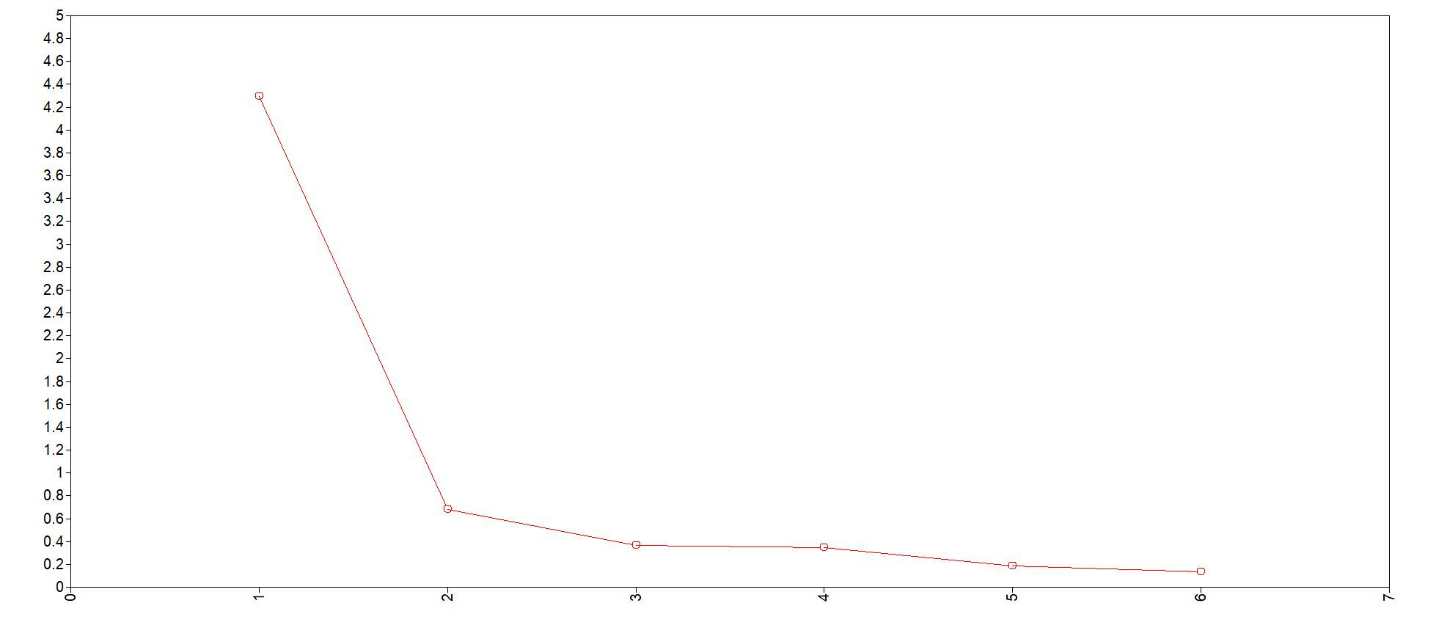


## Risky and Protective Dating Behaviors

Table 3. Eigenvalues for the sample correlation matrix, Risky and Protective Dating Behaviors EFA

|  | Full 14-item Scale* | | | 12-item Scale  (After Removing Items 2 and 5) | | |
| --- | --- | --- | --- | --- | --- | --- |
| Factor | Eigenvalue | Variance explained by factor | Total variance explained by solution | Eigenvalue | Variance explained by factor | Total variance explained by solution |
| 1 | 4.14 | 29.56% | 29.56% | 4.05 | 33.75% | 33.75% |
| 2 | 2.99 | 21.33% | 50.89% | 2.90 | 24.17% | 57.92% |
| 3 | 1.37 | 9.77% | 60.66% | 0.93 | 7.78% | 65.70% |
| 4 | 1.07 | 7.67% | 68.33% | 0.88 | 7.33% | 73.03% |
| 5 | 0.85 | 6.06% | 74.39% | 0.61 | 5.10% | 78.13% |
| 6 | 0.62 | 4.46% | 78.85% | 0.58 | 4.86% | 82.98% |
| 7 | 0.58 | 4.11% | 82.97% | 0.56 | 4.63% | 87.61% |
| 8 | 0.51 | 3.61% | 86.58% | 0.46 | 3.79% | 91.40% |
| 9 | 0.45 | 3.18% | 89.76% | 0.36 | 2.98% | 94.38% |
| 10 | 0.41 | 2.94% | 92.69% | 0.33 | 2.73% | 97.11% |
| 11 | 0.35 | 2.50% | 95.19% | 0.25 | 2.04% | 99.15% |
| 12 | 0.33 | 2.33% | 97.52% | 0.10 | 0.86% | 100.01% |
| 13 | 0.24 | 1.74% | 99.26% |  |  |  |
| 14 | 0.10 | 0.74% | 100.00% |  |  |  |
| Sum | 14.00 |  |  | 12.00 |  |  |

**Note*. While the Risky and Protective Dating Behaviors scale has 15 items, male respondents skipped one item so we consider the full scale for this research to be 14 items.

Figure 4. Scree Plot, Risky and Protective Dating Behaviors EFA, full 14-item scale


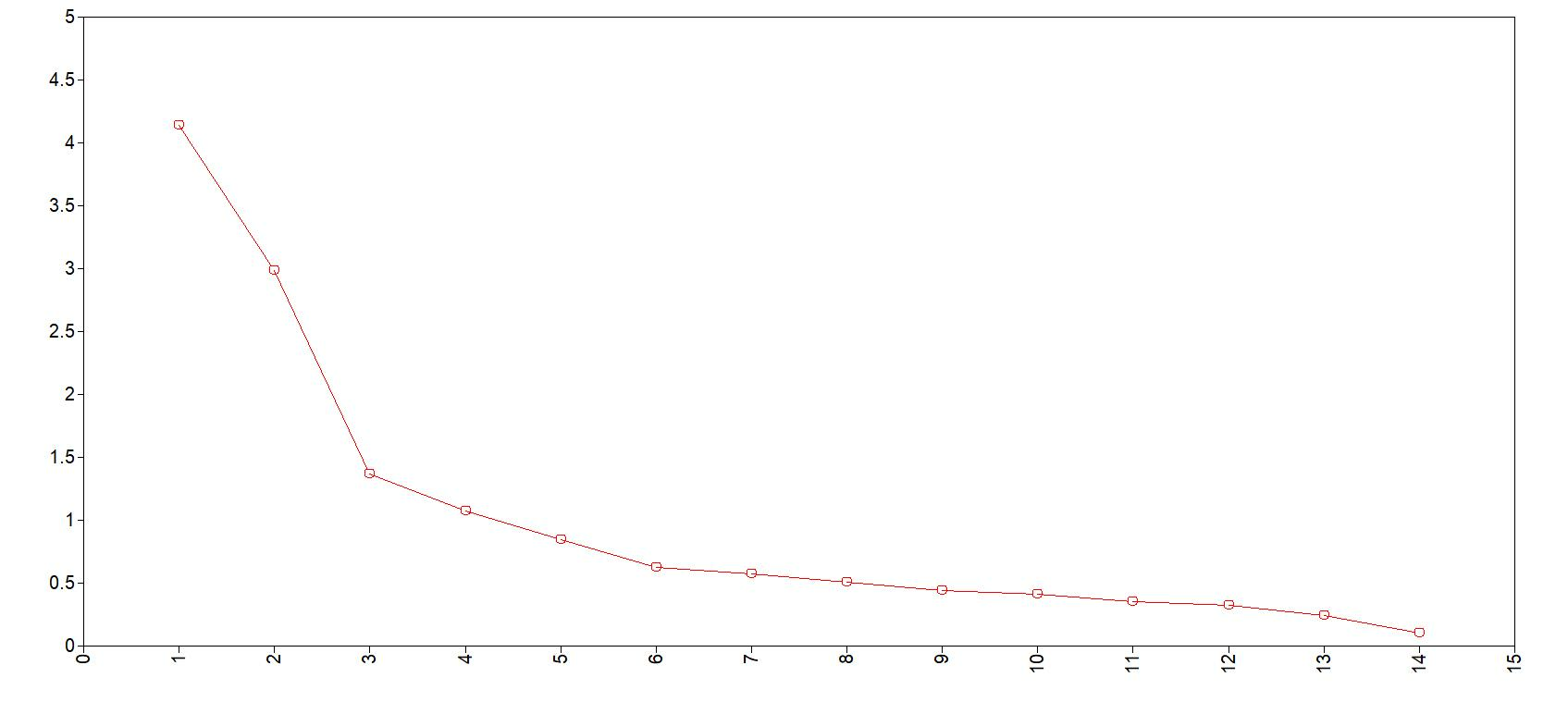


Figure 5. Scree Plot, Risky and Protective Dating Behaviors EFA, final 12-item scale after removing items 2 and 5


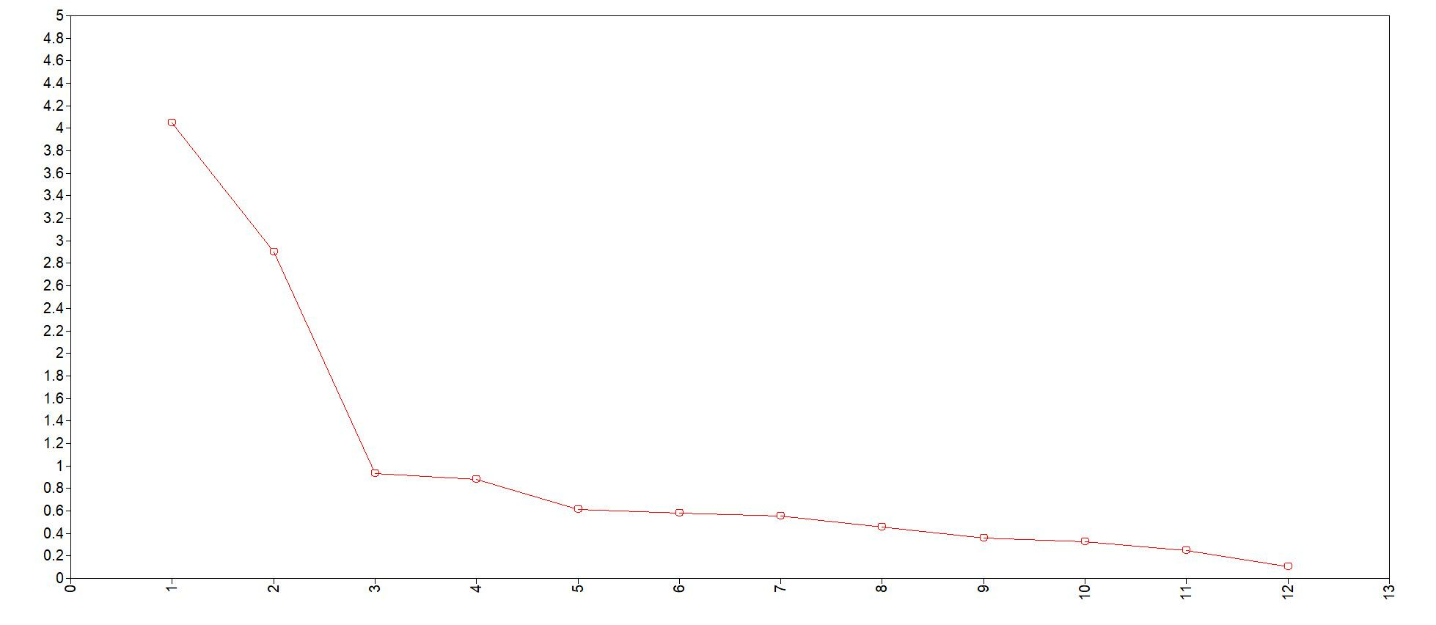


## Bystander Intentions

Table 4. Eigenvalues for the sample correlation matrix, Bystander Intentions EFA

|  | Full 11-item Scale | | | 8-item scale  (After removing reverse scored items: 5, 9, and 11) | | |
| --- | --- | --- | --- | --- | --- | --- |
| Factor | Eigenvalue | Variance explained by factor | Total variance explained by solution | Eigenvalue | Variance explained by factor | Total variance explained by solution |
| 1 | 5.41 | 49.22% | 49.22% | 5.40 | 67.49% | 67.49% |
| 2 | 1.91 | 17.40% | 66.62% | 0.84 | 10.53% | 78.01% |
| 3 | 0.90 | 8.22% | 74.84% | 0.42 | 5.20% | 83.21% |
| 4 | 0.55 | 4.97% | 79.81% | 0.37 | 4.61% | 87.83% |
| 5 | 0.48 | 4.36% | 84.18% | 0.31 | 3.89% | 91.71% |
| 6 | 0.41 | 3.76% | 87.93% | 0.25 | 3.06% | 94.78% |
| 7 | 0.36 | 3.29% | 91.23% | 0.22 | 2.79% | 97.56% |
| 8 | 0.31 | 2.79% | 94.02% | 0.20 | 2.44% | 100.00% |
| 9 | 0.24 | 2.21% | 96.23% |  |  |  |
| 10 | 0.22 | 2.02% | 98.25% |  |  |  |
| 11 | 0.19 | 1.75% | 100.00% |  |  |  |
| Sum | 11.00 |  |  | 8.00 |  |  |

Figure 6. Scree Plot, Bystander Intentions EFA, full 11-item scale


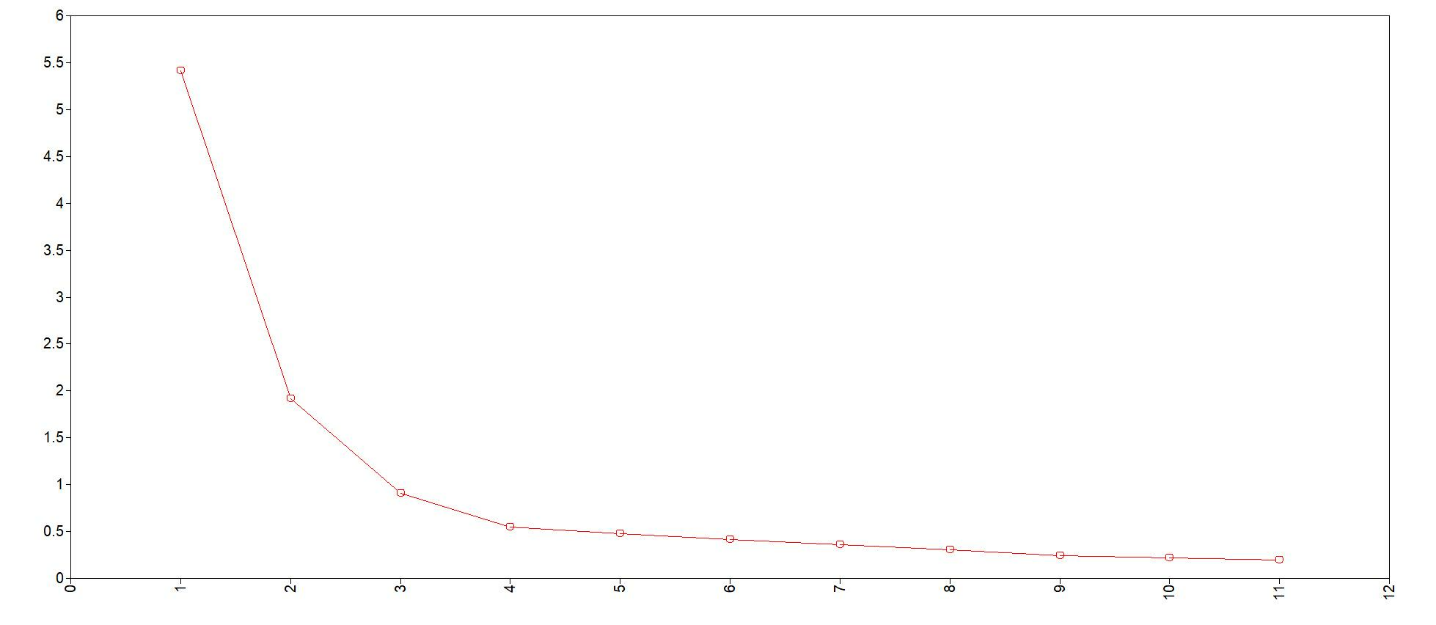


Figure 7. Scree plot, Bystander Intentions EFA, 8-item scale after removing reverse-scored items (Items 5, 9, and 11)


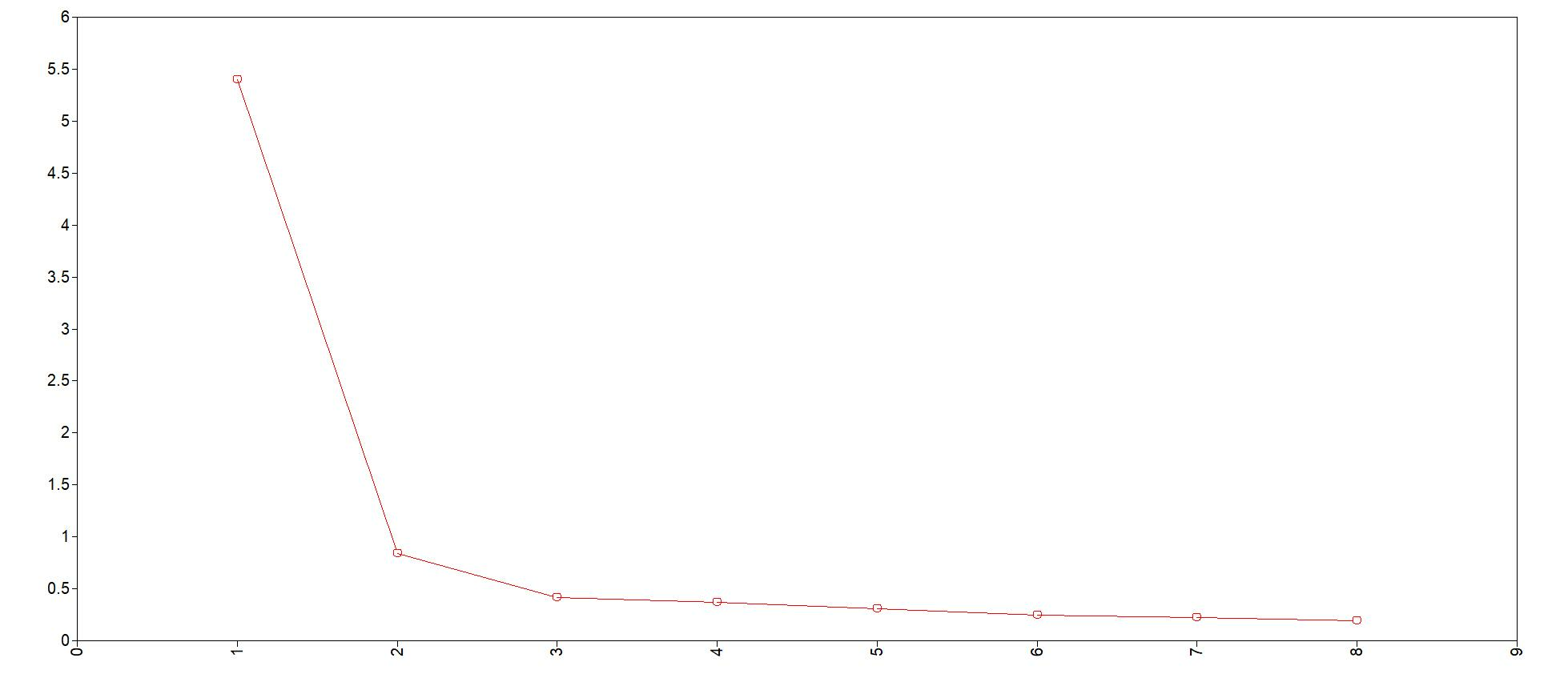

Supplement: S2 Appendix — (DOCX) [file pone.0317557.s002.docx]
