# Supplementary material for: Psychometric assessment of scales used to evaluate sexual assault prevention programming in the United States Air Force
Source: PLoS One. 2025 Jan 16;20(1):e0317557. doi: 10.1371/journal.pone.0317557 (PMC11737684; doi:10.1371/journal.pone.0317557)
Supplement: S3 Appendix — (DOCX) [file pone.0317557.s003.docx]

# S3 Appendix. Internal Consistency Reliability Overall, by Self-Identified Sex, and by SCC Program Assignment for Final Scales

## Summary – Cronbach’s Alpha, Final Measures

| Measure | # of items | Total Eligible Post-Training Sample | Males | Females | Female Revictimization | Female Primary Victimization | Male Revictimization | Male Primary Victimization | Male Healthy Relationships / Bystander Intervention |
| --- | --- | --- | --- | --- | --- | --- | --- | --- | --- |
| Consent-Related Assumptions | 4 | 0.71 | 0.72 | 0.60 | 0.57 | 0.62 | 0.64 | 0.61 | 0.74 |
| Rape Myths and Misconceptions | 4 | 0.86 | 0.88 | 0.75 | 0.74 | 0.76 | 0.76 | 0.84 | 0.89 |
| Self-Efficacy to Resist Unwanted Advances | 6 | 0.91 | 0.91 | 0.91 | 0.91 | 0.92 | 0.91 | 0.92 |  |
| Risky Dating Behaviors | 5 | 0.90 | 0.92 | 0.89 | 0.90 | 0.89 | 0.92 | 0.93 |  |
| Protective Dating Behaviors | 7 | 0.78 | 0.75 | 0.80 | 0.81 | 0.79 | 0.75 | 0.75 |  |
| Bystander Intentions | 8 | 0.93 |  |  |  |  |  |  | 0.93 |

## Consent Related Assumptions, Final 4-Item Scale

| Item | Overall Post-Training Sample (N=7,126) | | Males (N=5,301) | | Females (N=1,825) | | Female Revictimization (N=637) | | Female Primary Victimization (N=1,188) | | Male Revictimization (N=394) | | Male Primary Victimization (N=216) | | Male Healthy Relationships / Bystander Intervention (N=4,691) | |
| --- | --- | --- | --- | --- | --- | --- | --- | --- | --- | --- | --- | --- | --- | --- | --- | --- |
|  | Item-rest corr. | Alpha if Delete Item | Item-rest corr. | Alpha if Delete Item | Item-rest corr. | Alpha if Delete Item | Item-rest corr. | Alpha if Delete Item | Item-rest corr. | Alpha if Delete Item | Item-rest corr. | Alpha if Delete Item | Item-rest corr. | Alpha if Delete Item | Item-rest corr. | Alpha if Delete Item |
| 1. If someone is unsure about whether they want sex, it is okay for their partner to persist until they flatly say no. | 0.50 | 0.65 | 0.51 | 0.66 | 0.34 | 0.56 | 0.33 | 0.52 | 0.36 | 0.58 | 0.39 | 0.59 | 0.31 | 0.59 | 0.54 | 0.67 |
| 2. It is okay to have sex with someone who is drunk. | 0.54 | 0.62 | 0.57 | 0.62 | 0.40 | 0.51 | 0.37 | 0.48 | 0.42 | 0.54 | 0.45 | 0.55 | 0.42 | 0.51 | 0.59 | 0.63 |
| 3. If two people willingly go to some private or secluded place (such as one of their rooms), they intend to have sex. | 0.60 | 0.58 | 0.61 | 0.59 | 0.47 | 0.45 | 0.43 | 0.43 | 0.50 | 0.47 | 0.51 | 0.50 | 0.52 | 0.43 | 0.62 | 0.61 |
| 4. Both men and women alike see activities like kissing, touching, and fondling as a sign sexual intercourse is going to happen. | 0.34 | 0.73 | 0.33 | 0.75 | 0.30 | 0.59 | 0.28 | 0.55 | 0.32 | 0.61 | 0.33 | 0.63 | 0.31 | 0.59 | 0.34 | 0.77 |
| **Cronbach's Alpha** |  | **0.71** |  | **0.72** |  | **0.60** |  | **0.57** |  | **0.62** |  | **0.64** |  | **0.61** |  | **0.74** |

## Rape Myths and Misconceptions, Final 4-Item Scale

| Item | Overall Post-Training Sample (N=7,126) | | Males (N=5,301) | | Females (N=1,825) | | Female Revictimization (N=637) | | Female Primary Victimization (N=1,188) | | Male Revictimization (N=394) | | Male Primary Victimization (N=216) | | Male Healthy Relationships / Bystander Intervention (N=4,691) | |
| --- | --- | --- | --- | --- | --- | --- | --- | --- | --- | --- | --- | --- | --- | --- | --- | --- |
|  | Item-rest corr. | Alpha if Delete Item | Item-rest corr. | Alpha if Delete Item | Item-rest corr. | Alpha if Delete Item | Item-rest corr. | Alpha if Delete Item | Item-rest corr. | Alpha if Delete Item | Item-rest corr. | Alpha if Delete Item | Item-rest corr. | Alpha if Delete Item | Item-rest corr. | Alpha if Delete Item |
| 1. In many cases, if someone is raped by an acquaintance (someone they know), the person who was raped has to take some responsibility for what happened. | 0.80 | 0.88 | 0.80 | 0.90 | 0.45 | 0.74 | 0.43 | 0.73 | 0.46 | 0.75 | 0.45 | 0.76 | 0.64 | 0.80 | 0.60 | 0.91 |
| 2. If a person wants to increase their chances of having sex, they should get the other person drunk. | 0.87 | 0.81 | 0.88 | 0.83 | 0.61 | 0.65 | 0.63 | 0.62 | 0.60 | 0.67 | 0.54 | 0.72 | 0.64 | 0.80 | 0.79 | 0.83 |
| 3. When it comes to sex, women say no when they mean yes to avoid seeming "too easy." | 0.86 | 0.82 | 0.88 | 0.83 | 0.53 | 0.70 | 0.52 | 0.68 | 0.53 | 0.71 | 0.63 | 0.67 | 0.68 | 0.79 | 0.79 | 0.84 |
| 4. It is okay to have sex with someone who doesn't clearly communicate they want to have sex, as long as they aren't visibly resisting. (Note: communication can be verbal or non-verbal) | 0.88 | 0.80 | 0.90 | 0.82 | 0.59 | 0.67 | 0.52 | 0.68 | 0.63 | 0.66 | 0.64 | 0.66 | 0.67 | 0.79 | 0.82 | 0.82 |
| **Cronbach's Alpha** |  | **0.86** |  | **0.88** |  | **0.75** |  | **0.74** |  | **0.76** |  | **0.76** |  | **0.84** |  | **0.89** |

## Self-Efficacy to Resist Unwanted Advances, Final 6-Item Scale

| Item | Primary Victimization and Revictimization Prevention Sample  (N=2,435) | | Males (N=610) | | Females (N=1,825) | | Female Revictimization (N=637) | | Female Primary Victimization (N=1,188) | | Male Revictimization (N=394) | | Male Primary Victimization (N=216) | |
| --- | --- | --- | --- | --- | --- | --- | --- | --- | --- | --- | --- | --- | --- | --- |
|  | Item-rest corr. | Alpha if Delete Item | Item-rest corr. | Alpha if Delete Item | Item-rest corr. | Alpha if Delete Item | Item-rest corr. | Alpha if Delete Item | Item-rest corr. | Alpha if Delete Item | Item-rest corr. | Alpha if Delete Item | Item-rest corr. | Alpha if Delete Item |
| 1. Successfully resist someone's advances if they were attempting to get you to have sex and you were not interested? | 0.77 | 0.89 | 0.80 | 0.89 | 0.77 | 0.90 | 0.75 | 0.89 | 0.77 | 0.90 | 0.80 | 0.88 | 0.80 | 0.90 |
| 2. Tell someone that you would pay for your own way if they were attempting to pay for your meal when you did not want them to? | 0.66 | 0.91 | 0.68 | 0.91 | 0.65 | 0.91 | 0.61 | 0.91 | 0.68 | 0.92 | 0.68 | 0.90 | 0.68 | 0.92 |
| 3. Successfully resist someone's pressuring if they were attempting to get you to consume alcohol, despite your wishes not to do so? | 0.69 | 0.91 | 0.63 | 0.91 | 0.72 | 0.90 | 0.70 | 0.90 | 0.73 | 0.91 | 0.61 | 0.91 | 0.65 | 0.92 |
| 4. Successfully avoid a situation in which you could be sexually assaulted? | 0.81 | 0.89 | 0.85 | 0.88 | 0.80 | 0.89 | 0.80 | 0.88 | 0.80 | 0.90 | 0.85 | 0.87 | 0.84 | 0.90 |
| 5. Successfully think up ways to get out of a situation and execute your plan, if a situation develops in which you feel you could be in danger of sexual assault? | 0.82 | 0.89 | 0.80 | 0.89 | 0.83 | 0.89 | 0.83 | 0.88 | 0.83 | 0.89 | 0.77 | 0.89 | 0.86 | 0.90 |
| 6. Successfully recognize the signs that you might be in danger of being sexually assaulted? | 0.79 | 0.89 | 0.78 | 0.89 | 0.79 | 0.89 | 0.77 | 0.89 | 0.80 | 0.90 | 0.76 | 0.89 | 0.83 | 0.90 |
| **Cronbach's Alpha** |  | **0.91** |  | **0.91** |  | **0.91** |  | **0.91** |  | **0.92** |  | **0.91** |  | **0.92** |

## Risky Dating Behaviors, Final 5-Item Scale

| Item | Primary Victimization and Revictimization Prevention Sample with Dating History (N=1,797) | | Males with Dating History (N=478) | | Females with Dating History (N=1,319) | | Female Revictimization with Dating History (N=526) | | Female Primary Victimization with Dating History (N=793) | | Male Revictimization with Dating History (N=317) | | Male Primary Victimization with Dating History (N=161) | |
| --- | --- | --- | --- | --- | --- | --- | --- | --- | --- | --- | --- | --- | --- | --- |
|  | Item-rest corr. | Alpha if Delete Item | Item-rest corr. | Alpha if Delete Item | Item-rest corr. | Alpha if Delete Item | Item-rest corr. | Alpha if Delete Item | Item-rest corr. | Alpha if Delete Item | Item-rest corr. | Alpha if Delete Item | Item-rest corr. | Alpha if Delete Item |
| 1. I consume alcohol or other drugs | 0.78 | 0.88 | 0.84 | 0.90 | 0.76 | 0.87 | 0.78 | 0.87 | 0.72 | 0.86 | 0.83 | 0.90 | 0.85 | 0.90 |
| 2. My date consumes alcohol or other drugs | 0.81 | 0.87 | 0.85 | 0.90 | 0.80 | 0.86 | 0.81 | 0.86 | 0.78 | 0.85 | 0.87 | 0.89 | 0.82 | 0.91 |
| 3. I consume enough alcohol or other drugs to become drunk or high | 0.85 | 0.86 | 0.90 | 0.88 | 0.82 | 0.85 | 0.82 | 0.86 | 0.81 | 0.84 | 0.90 | 0.88 | 0.90 | 0.89 |
| 4. My date consumes enough alcohol or other drugs to become drunk or high | 0.81 | 0.87 | 0.86 | 0.89 | 0.78 | 0.86 | 0.79 | 0.86 | 0.76 | 0.85 | 0.87 | 0.89 | 0.84 | 0.91 |
| 5. I have "blacked out" from alcohol or other drugs (lose consciousness, can't remember what happened) | 0.56 | 0.92 | 0.56 | 0.95 | 0.55 | 0.91 | 0.53 | 0.92 | 0.55 | 0.90 | 0.52 | 0.95 | 0.64 | 0.94 |
| **Cronbach's Alpha** |  | **0.90** |  | **0.92** |  | **0.89** |  | **0.90** |  | **0.89** |  | **0.92** |  | **0.93** |

## Protective Dating Behaviors, Final 7-Item Scale

| Item | Primary Victimization and Revictimization Prevention Sample with Dating History (N=1,797) | | Males with Dating History (N=478) | | Females with Dating History (N=1,319) | | Female Revictimization with Dating History (N=526) | | Female Primary Victimization with Dating History (N=793) | | Male Revictimization with Dating History (N=317) | | Male Primary Victimization with Dating History (N=161) | |
| --- | --- | --- | --- | --- | --- | --- | --- | --- | --- | --- | --- | --- | --- | --- |
|  | Item-rest corr. | Alpha if Delete Item | Item-rest corr. | Alpha if Delete Item | Item-rest corr. | Alpha if Delete Item | Item-rest corr. | Alpha if Delete Item | Item-rest corr. | Alpha if Delete Item | Item-rest corr. | Alpha if Delete Item | Item-rest corr. | Alpha if Delete Item |
| 1. I provide my own transportation or carry enough money in case I need to get myself home later (e.g., for a bus, taxi, etc.) | 0.36 | 0.79 | 0.40 | 0.74 | 0.42 | 0.79 | 0.41 | 0.80 | 0.42 | 0.78 | 0.46 | 0.72 | 0.34 | 0.75 |
| 2. My date and I choose group activities (e.g., double date or spend time with friends) | 0.34 | 0.79 | 0.48 | 0.71 | 0.34 | 0.80 | 0.41 | 0.80 | 0.30 | 0.80 | 0.30 | 0.75 | 0.32 | 0.75 |
| 3. Before I go out with someone for the first time, I try to find out about them | 0.53 | 0.75 | 0.47 | 0.72 | 0.53 | 0.77 | 0.53 | 0.78 | 0.53 | 0.76 | 0.44 | 0.72 | 0.58 | 0.69 |
| 4. I pay attention to my date's alcohol or other drug intake | 0.51 | 0.76 | 0.50 | 0.71 | 0.52 | 0.77 | 0.49 | 0.79 | 0.54 | 0.76 | 0.50 | 0.71 | 0.38 | 0.73 |
| 5. My date and I meet in public places (e.g., a restaurant) | 0.59 | 0.74 | 0.52 | 0.70 | 0.60 | 0.76 | 0.62 | 0.77 | 0.58 | 0.75 | 0.48 | 0.72 | 0.58 | 0.69 |
| 6. I let a friend or family member know where I am and whom I am with | 0.61 | 0.74 | 0.54 | 0.70 | 0.63 | 0.75 | 0.66 | 0.76 | 0.62 | 0.75 | 0.51 | 0.71 | 0.55 | 0.70 |
| 7. In general, I plan for what self-protective measures I would take if I were alone with my date and they became sexually aggressive | 0.62 | 0.74 | 0.40 | 0.74 | 0.63 | 0.75 | 0.64 | 0.76 | 0.63 | 0.74 | 0.53 | 0.70 | 0.53 | 0.70 |
| **Cronbach's Alpha** |  | **0.78** |  | **0.75** |  | **0.80** |  | **0.81** |  | **0.79** |  | **0.75** |  | **0.75** |

## Bystander Intentions, Final 8-Item Scale

| Item | Male Healthy Relationships / Bystander Intervention Sample (N=4,691) | |
| --- | --- | --- |
|  | Item-rest corr. | Alpha if Delete Item |
| 1. Intervene if you saw a man hitting on a woman and she appeared not to want it | 0.76 | 0.92 |
| 2. Intervene if you witnessed a situation in which it looked like a woman might end up being taken advantage of | 0.84 | 0.91 |
| 3. Express discomfort if you heard a man/group of men using bad language or offensive names when talking about women | 0.64 | 0.93 |
| 4. Intervene if you saw a man hitting on a woman who appeared to be extremely intoxicated | 0.83 | 0.92 |
| 5. Express your discomfort if someone said that rape victims are to blame for being raped | 0.72 | 0.92 |
| 6. Ask a friend if they needed to be walked or driven home from a party | 0.66 | 0.93 |
| 7. Do something to help a drunk person who was being taken upstairs to a bedroom at a party | 0.82 | 0.92 |
| 8. Intervene if you saw a friend taking a very intoxicated person up the stairs to his/her room | 0.79 | 0.92 |
| **Cronbach's Alpha** |  | **0.93** |
