# Supplementary material for: Psychometric assessment of scales used to evaluate sexual assault prevention programming in the United States Air Force
Source: PLoS One. 2025 Jan 16;20(1):e0317557. doi: 10.1371/journal.pone.0317557 (PMC11737684; doi:10.1371/journal.pone.0317557)
Supplement: S4 Appendix — (DOCX) [file pone.0317557.s004.docx]

# S4 Appendix: Summary of EFA, CFA, and Internal Consistency Reliability Results

|  |  |  | Date Rape Attitudes | Self-Efficacy to Resist Unwanted Advances | Risky and Protective Dating Behaviors | Bystander Intentions |
| --- | --- | --- | --- | --- | --- | --- |
| EFA | # of observations in EFA (% eligible sample) | | 2,871 (80.6%) | 990 (81.3%) | 830 (92.0%) | 1,995 (85.0%) |
|  | # items in full, original scale | | 10 | 6 | 14 | 11 |
|  | Items identified as problematic in full-scale EFA | | Item 8 | None | Items 2 and 5 | Items 5, 9, and 11 |
|  | # of items included in final EFA | | 9 of 10 | 6 of 6 | 12 of 14 | 8 of 11 |
|  | EFA suggested # of factors | | 2 | 1 | 2 | 1 |
|  | Factor 1 | Name | Consent-Related Assumptions | Self-Efficacy Ratings | Risk Behaviors | Bystander Intentions |
|  |  | Eigenvalue | 4.26 | 4.29 | 4.05 | 5.40 |
|  |  | % variance explained by factor | 47.32% | 71.53% | 33.75% | 67.49% |
|  | Factor 2 | Name | Rape Myths & Misconceptions | NA | Protective Behaviors | NA |
|  |  | Eigenvalue | 1.12 | 0.68 | 2.9 | 0.84 |
|  |  | % variance explained by factor | 12.40% | 11.32% | 24.17% | 10.53% |
| CFA | # of observations in CFA (% eligible sample) | | 2,882 (80.9%) | 954 (78.4%) | 818 (91.4%) | 2,013 (85.8%) |
|  | # factors ran in CFA based on EFA results | | 2 | 1 | 2 | 1 |
|  | Items identified as problematic in CFA | | Item 5 | None | None | None |
|  | # items included in final CFA | | 8 Items | 6 Items | 12 Items | 8 Items |
|  | RMSEA | | 0.05 | 0.11 | 0.09 | 0.10 |
|  | CFI | | 0.97 | 0.90 | 0.85 | 0.91 |
|  | SRMR | | 0.03 | 0.06 | 0.05 | 0.05 |
|  | Satorra-Bentler Chi-Squared | | 129.73** | 37.41** | 412.94** | 3.09 |
| Cronbach’s Alpha (among total eligible sample) | | | 0.71; 0.86 | 0.91 | 0.90; 0.78 | 0.93 |
| Recommendations | | | Use the two 4-item subscales among male SCC participants only | Use the full 6-item scale among all victimization and revictimization SCC participants | Use only the 7-item Protective Behaviors subscale among victimization and revictimization SCC participants | Use the final 8-item scale among Healthy Relationships/ Bystander Intervention SCC participants |
